# Cereulide Synthetase Acquisition and Loss Events within the Evolutionary History of Group III *Bacillus cereus Sensu Lato* Facilitate the Transition between Emetic and Diarrheal Foodborne Pathogens

**DOI:** 10.1128/mBio.01263-20

**Published:** 2020-08-25

**Authors:** Laura M. Carroll, Martin Wiedmann

**Affiliations:** a Department of Food Science, Cornell University, Ithaca, New York, USA; University of Queensland

**Keywords:** *Bacillus* Emeticus, *Bacillus cereus*, emetic *Bacillus cereus*, cereulide, foodborne illness, genomic epidemiology, phylogenomics

## Abstract

Cereulide-producing members of Bacillus cereus *sensu lato* group III (also known as emetic B. cereus) possess cereulide synthetase, a plasmid-encoded, nonribosomal peptide synthetase encoded by the *ces* gene cluster. Despite the documented risks that cereulide-producing strains pose to public health, the level of genomic diversity encompassed by emetic B. cereus has never been evaluated at a whole-genome scale. Here, we employ a phylogenomic approach to characterize group III B. cereus *sensu lato* genomes which possess *ces* (*ces* positive) alongside their closely related, *ces*-negative counterparts (i) to assess the genomic diversity encompassed by emetic B. cereus and (ii) to identify potential *ces* loss and/or gain events within the evolutionary history of the high-risk and medically relevant sequence type (ST) 26 lineage often associated with emetic foodborne illness. Using all publicly available *ces*-positive group III B. cereus *sensu lato* genomes and the *ces*-negative genomes interspersed among them (*n* = 159), we show that emetic B. cereus is not clonal; rather, multiple lineages within group III harbor cereulide-producing strains, all of which share an ancestor incapable of producing cereulide (posterior probability = 0.86 to 0.89). Members of ST 26 share an ancestor that existed circa 1748 (95% highest posterior density [HPD] interval = 1246.89 to 1915.64) and first acquired the ability to produce cereulide before 1876 (95% HPD = 1641.43 to 1946.70). Within ST 26 alone, two subsequent *ces* gain events were observed, as well as three *ces* loss events, including among isolates responsible for B. cereus *sensu lato* toxicoinfection (i.e., “diarrheal” illness).

## Abstract (precis)

B. cereus is responsible for thousands of cases of foodborne disease each year worldwide, causing two distinct forms of illness: (i) intoxication via cereulide (i.e., emetic syndrome) or (ii) toxicoinfection via multiple enterotoxins (i.e., diarrheal syndrome). Here, we show that emetic B. cereus is not a clonal, homogenous unit that resulted from a single cereulide synthetase gain event followed by subsequent proliferation; rather, cereulide synthetase acquisition and loss is a dynamic, ongoing process that occurs across lineages, allowing some group III B. cereus *sensu lato* populations to oscillate between diarrheal and emetic foodborne pathogens over the course of their evolutionary histories. We also highlight the care that must be taken when selecting a reference genome for whole-genome sequencing-based investigation of emetic B. cereus *sensu lato* outbreaks, since some reference genome selections can lead to a confounding loss of resolution and potentially hinder epidemiological investigations.

## INTRODUCTION

The Bacillus cereus group, also known as B. cereus *sensu lato*, is a complex of closely related, Gram-positive, spore-forming lineages, which vary in their ability to cause illness in humans (1). Members of B. cereus *sensu lato* were estimated to be responsible for more than 256,000 foodborne intoxications worldwide in 2010 (2), although this is likely an underestimate due to the mild symptoms frequently associated with this illness (1). Foodborne B. cereus intoxication (i.e., “emetic” illness) is caused by cereulide, a highly heat- and pH-stable toxin, which is preformed in food prior to consumption. These intoxications have a relatively short incubation period (typically 0.5 to 6 h) and are often accompanied by symptoms of vomiting and nausea (1, 3–5). This can be contrasted with B. cereus toxicoinfection (i.e., “diarrheal” illness), a different form of illness in which multiple enterotoxins produced within the host small intestine yield diarrheal symptoms which typically onset after 8 to 16 h (1, 6). Notably, emetic and diarrheal symptoms are not always congruent with B. cereus emetic and diarrheal syndromes, respectively, as both vomiting and diarrhea may be reported among cases (7, 8).

Production of cereulide, the toxin responsible for emetic B. cereus foodborne illness, can be attributed to cereulide synthetase, a nonribosomal peptide synthetase encoded by the cereulide synthetase biosynthetic gene cluster (*ces*) (9, 10). *ces* has been detected in two major B. cereus *sensu lato* phylogenetic groups (assigned using the sequence of pantoate-beta-alanine ligase [*panC*] and a seven-group typing scheme): group III and group VI (10–16). While cereulide-producing group VI strains, also known as “emetic B. weihenstephanensis,” have been isolated on rare occasions (14, 15, 17–19), the bulk of cereulide-producing strains belong to group III (8, 10, 13, 16). Often referred to as “emetic B. cereus,” cereulide-producing group III strains often harbor *ces* on plasmids (9, 10, 19) and have been linked to outbreaks around the world (5, 7, 8, 20). It is essential to note that group III B. cereus *sensu lato* isolates do not belong to the B. cereus *sensu stricto* species (i.e., B. cereus *sensu lato* group IV) (7, 21).

Despite the documented risks that cereulide-producing strains pose to public health, the level of diversity encompassed by emetic B. cereus has not been evaluated at a whole-genome scale. Furthermore, potential heterogeneity in cereulide production capabilities among lineages encompassed by emetic B. cereus has never been assessed; plasmid-encoded *ces*, and thus the ability to produce cereulide, can hypothetically be gained or lost within a lineage, although the extent to which this happens is unknown. Here, we employ phylogenomic approaches to characterize group III B. cereus *sensu lato* genomes that possess *ces* (*ces* positive) alongside their closely related *ces*-negative counterparts (i) to assess the genomic diversity encompassed by cereulide-producing group III strains (i.e., emetic B. cereus) and (ii) to identify potential *ces* loss and/or gain events within the emetic B. cereus evolutionary history.

## RESULTS

### Cereulide-producing group III *B. cereus sensu lato* strains are distributed across multiple lineages and share an ancestor incapable of synthesizing cereulide.

**FIG 1:**
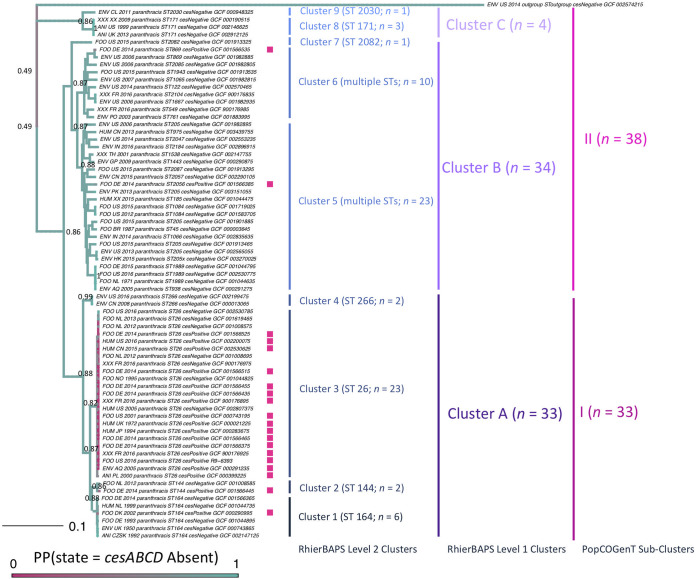
Maximum-likelihood phylogeny constructed using core SNPs identified among 71 emetic group III B. cereus *sensu lato* genomes and their closely related, nonemetic counterparts, plus outgroup genome B. cereus *sensu lato* strain AFS057383. Tip labels of genomes that possess cereulide synthetase-encoding genes *cesABCD* are annotated by a pink square. Clade labels correspond to (i) RhierBAPs level 2 cluster assignments, denoted as clusters 1 to 9, with the number of isolates assigned to a cluster (*n*) and sequence type (ST) determined using *in silico* multilocus sequence typing (MLST) listed in parentheses; (ii) RhierBAPs level 1 cluster assignments, denoted as clusters A, B, and C; (iii) PopCOGenT subcluster assignments, denoted as I and II. Tree edge and node colors correspond to the posterior probability (PP) of being in a *ces-*negative state, obtained using an empirical Bayes approach, in which a continuous-time reversible Markov model was fitted, followed by 1,000 simulations of stochastic character histories using the fitted model and tree tip states. Equal root node prior probabilities for *ces*-positive and *ces*-negative states were used. Node labels denote selected PP values, chosen for readability. The phylogeny was rooted along the outgroup genome, and branch lengths are reported in substitutions/site.

Overall, 31 sequence types (STs) assigned using *in silico* multilocus sequence typing (MLST) were observed among 159 group III isolates included in this study, with genomes that possessed *cesABCD* (referred to here as “*ces*-positive” genomes) represented by five STs (ST 26, 144, 164, 869, and 2056; Fig. 1 and see Table S1 in the supplemental material). Four of these STs (ST 26, 144, 164, and 869) also encompassed one or more isolates that lacked cereulide synthetase (referred to here as “*ces*-negative isolates”; Fig. 1 and see Table S1).

10.1128/mBio.01263-20.2 TABLE S1 Genomic data and metadata used in this study (*n* = 159). Download Table S1, XLSX file, 0.03 MB. Copyright © 2020 Carroll and Wiedmann. 2020 Carroll and Wiedmann This content is distributed under the terms of the Creative Commons Attribution 4.0 International license.

**FIG 2:**
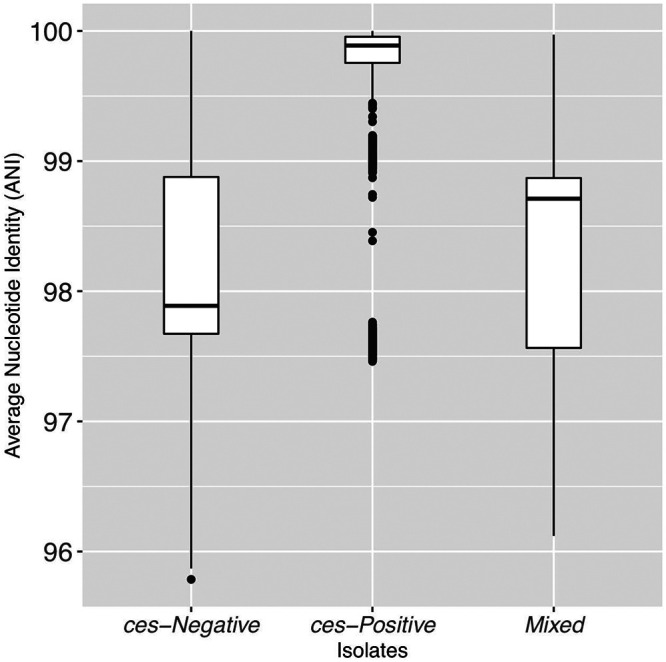
Pairwise ANI values calculated between 159 group III B. cereus *sensu lato* genomes in which (i) both the query and reference genome lacked *cesABCD* (*ces* negative; *n* = 94); (ii) both the query and reference genome possessed *cesABCD* (*ces* positive; *n* = 65); and (iii) the query genome possessed *cesABCD* and the reference genome lacked *cesABCD* and vice versa (mixed). Pairwise ANI values were calculated using FastANI v1.0. Lower and upper box hinges correspond to the first and third quartiles, respectively. Lower and upper whiskers extend from the hinge to the smallest and largest values no more distant than 1.5 times the interquartile range from the hinge, respectively. Points represent pairwise distances that fall beyond the ends of the whiskers.

The group III genomes queried here were distributed into three major clusters and nine subclusters using RhierBAPs, with *ces*-positive isolates present in two and five clusters and subclusters, respectively (Fig. 1). When PopCOGenT was used to delineate populations using recent gene flow, genomes were distributed among two subclusters (i.e., populations), with *ces*-positive genomes present in both subclusters. All genomes were assigned to a single “main cluster,” a unit that has been proposed to mirror the “species” definition applied to plants and animals (Fig. 1) (22). Congruent with these findings, average nucleotide identity (ANI) values calculated between genomes confirmed that all cereulide-producing group III strains are members of the same genomospecies using any previously proposed genomospecies threshold for B. cereus *sensu lato* (i.e., 92.5 to 96 ANI) (21, 23–26). However, considerable genomic diversity existed among cereulide-producing isolates, as *ces*-positive genomes could share as low as 97.5 ANI with others (Fig. 2).

The common ancestor of all *ces*-positive group III genomes was predicted to not possess *ces*, regardless of outgroup, use of core or majority single nucleotide polymorphisms (SNPs), or root prior (*ces-*negative state posterior probability [PP] 0.86 to 0.89; Fig. 1, Fig. S1 and S2 [https://doi.org/10.6084/m9.figshare.c.5057276.v1], and Table S2). For STs 144, 164, 869, and 2056, a single *ces*-positive isolate was present among genomes assigned to the ST (Fig. 1). Consequently, a single acquisition event was predicted to be responsible for the presence of *ces*-positive lineages within each of these STs, and the common ancestor shared by each ST encompassing more than one genome was predicted to lack *ces* (Fig. 1, Fig. S1 and S2 [https://doi.org/10.6084/m9.figshare.c.5057276.v1], and Table S2). The common ancestor shared by ST 26 was predicted to not possess *ces* when core SNPs were used for phylogeny construction (*ces*-negative PP 0.78 to 0.83, depending on choice of outgroup; Fig. 1, Fig. S1 and S2, and Table S2); however, the inclusion of noncore SNPs (i.e., all or majority SNPs) and choice of clusters used for ancestral state reconstruction led to greater uncertainty (*ces*-negative PP 0.10 to 0.61; Table S2).

10.1128/mBio.01263-20.3 TABLE S2 Results of cereulide synthetase ancestral state reconstruction for maximum-likelihood (ML) phylogenies. Download Table S2, XLSX file, 0.01 MB. Copyright © 2020 Carroll and Wiedmann. 2020 Carroll and Wiedmann This content is distributed under the terms of the Creative Commons Attribution 4.0 International license.

### ST 26 first acquired the ability to produce cereulide prior to 1876.

**FIG 3:**
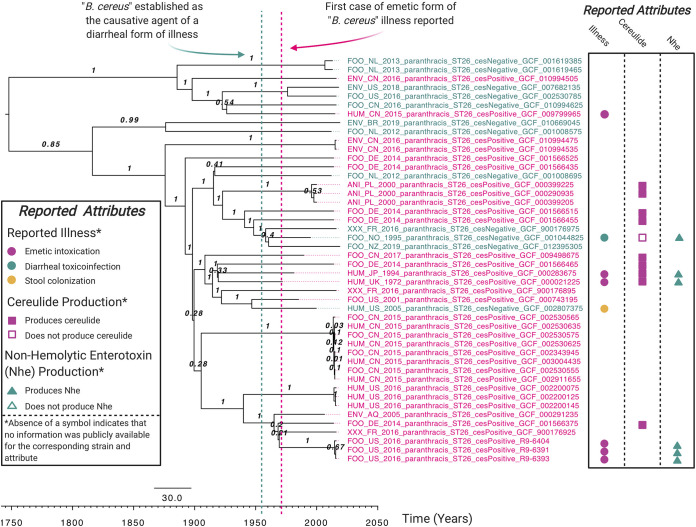
Rooted, time-scaled maximum clade credibility (MCC) phylogeny constructed using core SNPs identified among 46 group III B. cereus *sensu lato* genomes assigned to sequence type (ST) 26. Isolation years for strains ranged from 1972 to 2019 (Table S3). Tip label colors denote *ces*-positive (pink) and *ces*-negative (teal) genomes predicted to be capable and incapable, respectively, of producing cereulide. Tip labels of isolates that could be associated with one or more of the following attributes using publicly available metadata are annotated on the right side with symbols displayed in the “Reported Attributes” legend: (i) a known B. cereus *sensu lato* illness (emetic intoxication, diarrheal toxicoinfection, or stool colonization), (ii) cereulide-producing phenotype (reported to produce cereulide or not; note that strains could not merely possess or lack *ces* genes, but had to be reported as cereulide-producing or not cereulide-producing using phenotypic methods), and/or (iii) nonhemolytic enterotoxin (Nhe)-producing phenotype (reported to produce Nhe or not; note that strains could not merely possess or lack *nhe* genes, but had to be reported as Nhe-producing or not Nhe-producing using phenotypic methods). The absence of a symbol indicates that no information was publicly available for the corresponding strain and attribute (e.g., additional isolates were associated with illness; however, these are not annotated, since the type of illness could not be confirmed from the available literature). Branch labels denote posterior probabilities of branch support. Time in years is plotted along the *x* axis, with branch lengths reported in years. Core SNPs were identified using Snippy v4.3.6. The phylogeny was constructed using the results of five independent runs using a relaxed lognormal clock model, the Standard_TVMef nucleotide substitution model, and the Coalescent Bayesian Skyline population model implemented in BEAST v2.5.1, with 10% burn-in applied to each run. LogCombiner-2 was used to combine BEAST 2 log files, and TreeAnnotator-2 was used to construct the phylogeny using median node heights. The figure was annotated using BioRender. See Fig. S3 (https://doi.org/10.6084/m9.figshare.c.5057276.v1) to view node height 95% highest posterior density (HPD) intervals.

**FIG 4:**
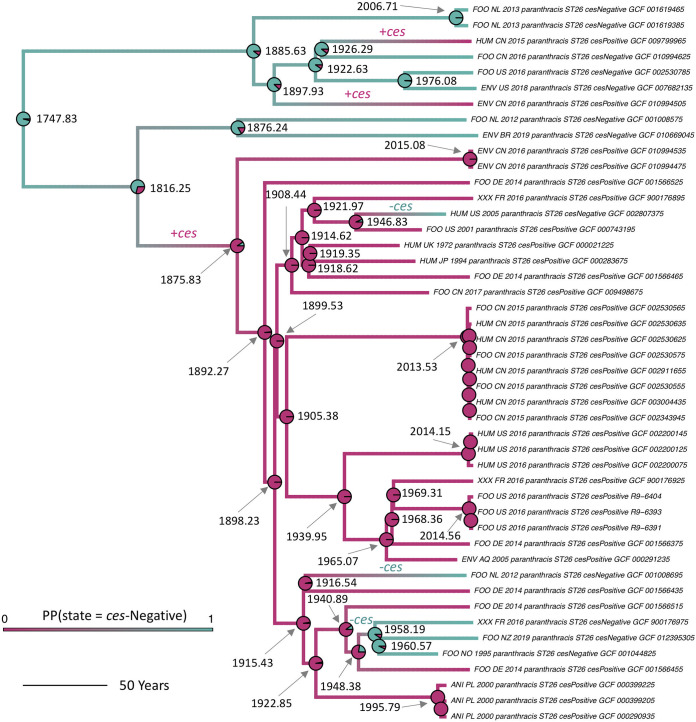
Rooted, time-scaled maximum clade credibility (MCC) phylogeny constructed using core SNPs identified among 46 group III B. cereus *sensu lato* genomes assigned to sequence type (ST) 26. Branch color corresponds to posterior density, denoting the probability of a lineage being in a *ces*-negative state as determined using ancestral state reconstruction. Pie charts at nodes denote the posterior probability (PP) of a node being in a *ces*-negative (teal) or *ces*-positive (pink) state. Labels along branches denote a *ces* gain or loss event (denoted by +*ces* or –*ces*, respectively). Node labels indicate node ages in years (selected for readability) and are either placed to the right of their corresponding node pie chart or connected to their corresponding node pie chart with a gray arrow. Branch lengths are reported in years. Core SNPs were identified using Snippy v4.3.6. The phylogeny was constructed using the results of five independent runs using a relaxed lognormal clock model, the Standard_TVMef nucleotide substitution model, and the Coalescent Bayesian Skyline population model implemented in BEAST v2.5.1, with 10% burn-in applied to each run. LogCombiner-2 was used to combine BEAST 2 log files, and TreeAnnotator-2 was used to construct the phylogeny using median node heights. Ancestral state reconstruction was performed using a prior on the root node in which the probability of the ST 26 ancestor being *ces* positive or *ces* negative was 0.2 and 0.8, respectively (see Tables S2 and S5 in the supplemental material). For ST 26 ancestral state reconstruction results obtained using different root node priors and isolate sets, see Table S5 and see Fig. S11 to S18 (https://doi.org/10.6084/m9.figshare.c.5057276.v1).

ST 26 was the only ST that encompassed multiple *ces*-negative and *ces*-positive strains (Fig. 1); therefore, the dynamics of cereulide synthetase loss and gain could be assessed among members of this lineage. ST 26 isolates in this study were predicted to evolve from a common ancestor that existed circa 1747.83 (95% highest posterior density interval [HPD] 1246.89 to 1915.64; Fig. 3 and Fig. S3 to S10 [https://doi.org/10.6084/m9.figshare.c.5057276.v1]) with an estimated evolutionary rate of 1.52 × 10^−7^ substitutions/site/year (95% HPD 3.55 × 10^−8^ to 2.59 × 10^−7^ substitutions/site/year; see Tables S3 and S4 in the supplemental material). *ces* was predicted to have been first acquired within ST 26 prior to ≈1876 (95% HPD 1641.43 to 1946.70, respectively; Fig. 3 and 4, Fig. S11 to S18 [https://doi.org/10.6084/m9.figshare.c.5057276.v1], and Table S5). Two subsequent *ces* gain events within ST 26 were observed: (i) one after 1897.93 (95% HPD 1692.71 to 1965.73), and (ii) a second after 1926.29 (95% HPD 1775.85 to 1987.95). *ces* loss events among ST 26 were predicted to have occurred on three occasions: (i) after 1916.54 (95% HPD 1758.18 to 1965.89), (ii) after 1946.83 (95% HPD 1850.98 to 1982.83), and (iii) between 1948.38 and 1958.19 (95% HPD 1832.50 to 1985.02 and 1856.69 to 1990.36, respectively; Fig. 3 and 4, Fig. S11 to S18 [https://doi.org/10.6084/m9.figshare.c.5057276.v1], and Table S5) (16).

10.1128/mBio.01263-20.4 TABLE S3 Extended summary of temporal analyses for various B. cereus *sensu lato* ST 26 data sets. Download Table S3, XLSX file, 0.01 MB. Copyright © 2020 Carroll and Wiedmann. 2020 Carroll and Wiedmann This content is distributed under the terms of the Creative Commons Attribution 4.0 International license.

10.1128/mBio.01263-20.5 TABLE S4 Model selection results for various B. cereus *sensu lato* ST 26 clock/population model combinations. Download Table S4, XLSX file, 0.01 MB. Copyright © 2020 Carroll and Wiedmann. 2020 Carroll and Wiedmann This content is distributed under the terms of the Creative Commons Attribution 4.0 International license.

10.1128/mBio.01263-20.6 TABLE S5 Results of cereulide synthetase ancestral state reconstruction for ST 26 Bayesian phylogenies. Download Table S5, XLSX file, 0.01 MB. Copyright © 2020 Carroll and Wiedmann. 2020 Carroll and Wiedmann This content is distributed under the terms of the Creative Commons Attribution 4.0 International license.

### Choice of emetic group III reference genome for reference-based SNP calling affects ST 26 phylogenomic topology.

SNP identification using reference-based approaches and subsequent phylogeny construction are critical methods used in foodborne pathogen surveillance and outbreak investigation efforts. To determine whether choice of emetic reference genome could affect the topology of the ST 26 phylogeny, SNPs were identified among 64 ST 26 genomes using four reference-based SNP calling pipelines and six emetic reference genomes, which encompassed all observed group III emetic STs (Table 1). Notably, the emetic group III genome that was most distantly related to ST 26 (ST 869) did not yield sufficient resolution to produce a phylogeny when it was used as a reference for BactSNP/Gubbins and Snippy/Gubbins (Tables 1 and 2). For the BactSNP pipeline, the emetic ST 2056 genome additionally did not yield an alignment of SNPs among ST 26 isolates when it was used as a reference (Tables 1 and 2).

**TABLE 1:** Topological comparisons of B. cereus *sensu lato* ST 26 phylogenies constructed using various SNP calling pipeline/reference genome combinations

Reference genome					Kendall-Colijn test raw *P* values^*c*^			
Strain	NCBI RefSeq accession	Assembly level	MLST ST^*a*^	ANI range (mean)^*b*^	BactSNP	Lyve-SET	Parsnp	Snippy
AH187	NC_011658.1	Complete genome	26	99.8–100.0 (99.9)	0/0	0/0	0/0	0/0
IS195	GCF_000399225.1	Scaffold	26	99.6–100.0 (99.7)	0/0	0/0	0/0	0/0
AND1407	GCF_000290995.1	Scaffold	164	98.9–99.2 (99.1)	0/0	0/0	0/0	0/0
MB.17	GCF_001566445.1	Contigs	144	98.8–99.1 (99.0)	0/0	3.0 × 10^–4^/0	0/1.0	0/1.0
MB.18	GCF_001566385.1	Contigs	2056	97.4–97.8 (97.6)	NA^*d*^	2.0 × 10^–4^/0	0/0	0/0
MB.22	GCF_001566535.1	Contigs	869	97.4–97.7 (97.6)	NA^*e*^	3.0 × 10^–4^/0	0/0	NA^*e*^

a Seven-gene multilocus sequence typing (MLST) sequence types (ST) were determined *in silico* using BTyper v2.3.3.

b Range and mean average nucleotide identity (ANI) values were calculated between the respective reference genome and 64 group III B. cereus *sensu lato* genomes assigned to ST 26 using FastANI v1.0.

c Data are presented as “AH187 Reference Tree/AH187 Query Tree.” For each reference-based SNP calling pipeline (i.e., BactSNP, Lyve-SET, Parsnp, and Snippy), the phylogeny produced using SNPs identified among 64 B. cereus *sensu lato* ST 26 isolates using the respective SNP calling pipeline and the chromosome of B. cereus *sensu lato* ST 26 strain AH187 as a reference genome was used as a (i) reference tree and (ii) query tree for the Kendall-Colijn test, since the chromosome of strain AH187 has been shown to be an adequate reference genome for reference-based SNP calling among ST 26 genomes (7). NA, not applicable.

d No SNPs identified among the 64 B. cereus *sensu lato* ST 26 genomes using the respective SNP calling pipeline/reference genome combination.

e SNPs identified using the respective SNP calling pipeline/reference genome combination were not diverse enough for use with Gubbins/IQ-TREE.

**TABLE 2:** Pairwise SNP differences calculated between 64 B. cereus *sensu lato* ST 26 isolates, including 30 emetic isolates from a 2016 foodborne outbreak in New York State (NYS), using various SNP calling pipeline/reference genome combinations

SNP calling pipeline	Reference strain	MLST^*a*^	ANI range (mean)^*b*^	Range (median; mean)			Min/Max difference^*c*^
				Within NYS outbreak	Between NYS and non-NYS outbreaks	Within non-NYS outbreak	
BactSNP	AH187^*d*^	26	99.8–100.0 (99.9)	0–8 (2; 2.7)	58–381 (127; 149.6)	0–477 (162; 183.3)	50
	IS195	26	99.6–100.0 (99.7)	0–8 (2; 2.7)	58–385 (128; 153.9)	0–483 (167; 187.7)	50
	AND1407	164	98.9–99.2 (99.1)	0–8 (2; 2.7)	56–378 (125; 147.3)	0–472 (157; 178.1)	48
	MB.17	144	98.8–99.1 (99.0)	0 to 7 (2; 2.3)	57–370 (123; 144.3)	0–448 (153; 175.3)	50
	MB.18	2056	97.4–97.8 (97.6)	NA^*e*^	NA^*e*^	NA^*e*^	NA^*e*^
	MB.22	869	97.4–97.7 (97.6)	NA^*f*^	NA^*f*^	NA^*f*^	NA^*f*^
							
Lyve-SET	AH187^*d*^	26	99.8–100.0 (99.9)	0–7 (2; 2.6)	61–1,840 (169; 510.4)	0–2,246 (198; 520.9)	54
	IS195	26	99.6–100.0 (99.7)	0–6 (2; 2.3)	61–1,421 (174; 428.1)	0–1,834 (192; 447.7)	55
	AND1407	164	98.9–99.2 (99.1)	0–5 (2; 2.3)	56–1,622 (147; 449.0)	0–1,943 (167; 451.7)	51
	MB.17	144	98.8–99.1 (99.0)	0–5 (2; 2.0)	56–1,479 (144; 419.2)	0–1,830 (168; 429.7)	51
	MB.18	2056	97.4–97.8 (97.6)	0–4 (1; 1.6)	47–1,336 (114; 367.2)	0–1,578 (126; 363.0)	43
	MB.22	869	97.4–97.7 (97.6)	0–4 (1; 1.6)	44–1,323 (115; 363.3)	0–1,576 (127; 360.0)	40
							
Parsnp	AH187^*d*^	26	99.8–100.0 (99.9)	0–44 (9; 12.0)	59–2,404 (190; 697.4)	0–3,250 (260; 754.1)	15
	IS195	26	99.6–100.0 (99.7)	0–43 (9; 12.1)	62–2,414 (209; 705.3)	0–3,280 (269; 762)	19
	AND1407	164	98.9–99.2 (99.1)	0–44 (9; 11.8)	59–2,399 (185; 642.9)	0–2,832 (249–647.1)	15
	MB.17	144	98.8–99.1 (99.0)	0–42 (9; 11.8)	63–2,130 (189; 583.5)	0–2,527 (245; 585.9)	21
	MB.18	2056	97.4–97.8 (97.6)	0–41 (8; 10.6)	56–2,191 (170; 593)	0–2,551 (226; 596.3)	15
	MB.22	869	97.4–97.7 (97.6)	0–37 (8; 10.5)	57–2,180 (167; 595.1)	0–2,567 (227; 597.3)	20
							
Snippy	AH187^*d*^	26	99.8–100.0 (99.9)	0–7 (2; 2.6)	57–372 (146; 155.5)	0–444 (157; 177.6)	50
	IS195	26	99.6–100.0 (99.7)	0–7 (2; 2.6)	58–370 (145; 153.6)	0–436 (157; 176.2)	51
	AND1407	164	98.9–99.2 (99.1)	0–18 (5; 4.9)	55–368 (143; 152.9)	0–434 (156; 173)	37
	MB.17	144	98.8–99.1 (99.0)	0–20 (4; 4.4)	60–373 (138; 151.8)	0–436 (153; 171.9)	40
	MB.18	2056	97.4–97.8 (97.6)	0–50 (5; 9.3)	55–350 (128; 145.7)	0–401 (133; 159.5)	5
	MB.22	869	97.4–97.7 (97.6)	NA^*f*^	NA^*f*^	NA^*f*^	NA^*f*^

a Seven-gene multilocus sequence types (MLST) were determined *in silico* using BTyper v2.3.3.

b ANI range and mean values were calculated between the respective reference genome and 64 group III B. cereus *sensu lato* genomes assigned to ST 26 using FastANI v1.0.

c The Min/Max difference is the maximum number of SNPs identified between two outbreak isolates subtracted from the minimum number of SNPs between an outbreak and nonoutbreak isolate.

d AH187 has previously been shown to be an adequate reference genome for reference-based SNP calling among emetic ST 26 genomes (7).

e No SNPs identified among the 64 B. cereus *sensu lato* ST 26 genomes using the respective SNP calling pipeline/reference genome combination.

f SNPs identified using the respective SNP calling pipeline/reference genome combination were not diverse enough for use with Gubbins/IQ-TREE.

For the remaining SNP calling pipeline/reference genome combinations, the resulting phylogeny was compared to the phylogeny produced using the respective pipeline and the chromosome of ST 26 strain AH187 as a reference. In addition to being a well-characterized emetic strain for which a closed genome is available, strain AH187 was closely related to the 64 ST 26 isolates queried here and has previously been shown to serve as an adequate reference genome for SNP calling within ST 26 (7). For all SNP calling pipelines, phylogenies produced using the genomes of emetic ST 26 strain IS195 and emetic ST 164 strain AND1407 as references were more topologically similar to those produced using strain AH187 than would be expected by chance (Kendall-Colijn *P* < 0.05 after a Bonferroni correction; Table 1). However, the topology of phylogenies produced using Parsnp and Snippy with emetic ST 144 strain MB.17 differed from that produced using strain AH187 (Kendall-Colijn *P* > 0.05 after a Bonferroni correction; Table 1). Lyve-SET was the only pipeline that produced phylogenies that were more topologically similar to that produced using str. AH187 than would be expected by chance, regardless of emetic reference (Kendall-Colijn *P* < 0.05 after a Bonferroni correction; Table 1).

Despite producing phylogenies that resembled the AH187 phylogeny for five of six emetic reference genomes (Kendall-Colijn *P* < 0.05 after a Bonferroni correction; Table 1), core SNPs identified with Parsnp yielded relatively large pairwise SNP distances between emetic ST 26 genomes from a known outbreak (7). Regardless of reference genome selection, the difference between the minimum number of SNPs shared between outbreak and nonoutbreak isolates and the maximum number of SNPs detected between two outbreak isolates was less than the maximum number of SNPs shared between two outbreak isolates (Table 2). A similar phenomenon was observed when Snippy was used with a distant emetic ST 2056 strain as a reference (Table 2).

## DISCUSSION

### Group III *B. cereus sensu lato* isolates capable of causing emetic foodborne illness are not clonal.

Cereulide-producing B. cereus *sensu lato* strains are estimated to be responsible for thousands of cases of foodborne illness each year worldwide (2), including rare but severe forms of illness which may result in death (27–31). Although efforts to characterize this important pathogen using whole-genome sequencing have begun only recently, the amount of publicly available genomic data derived from emetic B. cereus has been increasing (21). Consequently, the current dogma regarding the evolutionary history of this group of organisms must be revisited. In early studies of cereulide-producing members of group III, *ces*-positive isolates were confined to a highly clonal complex within B. cereus *sensu lato* (10, 16); however, subsequent efforts have hinted that emetic B. cereus may showcase a considerably greater degree of genomic diversity than previously thought (21, 32–34).

Using all publicly available emetic group III B. cereus *sensu lato* genomes and the nonemetic genomes interspersed among them, we show on a whole-genome scale that emetic B. cereus is not clonal. Emetic toxin production capabilities within group III are not the result of a single cereulide synthetase gain event followed by subsequent proliferation; rather, the common ancestor of all cereulide-producing group III isolates was likely incapable of producing cereulide, and emetic toxin production capabilities resulted from at least seven independent cereulide synthetase acquisition events (at least one in each of STs 144, 164, 869, and 2056 and at least three in ST 26; Fig. 1 and 4). Pairwise ANI values calculated between emetic group III strains were as low as 97.5 ANI; for comparison, all members of the highly similar B. anthracis lineage commonly attributed to anthrax illness share ≥99.9 ANI with one another (21, 35), while genomes belonging to Salmonella enterica subsp. *enterica* (which is not considered to be clonal) can share pairwise ANI values as low as 97.0 (calculated between 425 genomes using FastANI as described in Materials and Methods) (36).

These findings are important, as unexpected diversity can confound bioinformatic analyses used to identify outbreaks from genomic data. For example, an evolutionarily distant reference genome can affect which SNPs are identified during reference-based SNP calling among bacterial genomes (7, 37–40). This can, in turn, affect metrics used to determine whether an isolate should be included or excluded from an outbreak (e.g., the topology of a resulting phylogeny, pairwise SNP cutoffs) (7, 38–40). Here, we showed that emetic group III isolates are considerably diverse, so much so that the use of some emetic B. cereus genomes as references for SNP calling can lead to a topologically confounding loss of resolution. The use of BactSNP/Gubbins and Snippy/Gubbins with distant emetic ST 869 as a reference, for example, yielded SNPs that could not reliably differentiate ST 26 genomes from each other. In an outbreak scenario, these approaches would incorrectly place nonoutbreak isolates among outbreak ones, potentially confounding an investigation. It is thus essential that the diversity of emetic B. cereus is acknowledged and accounted for to ensure that epidemiological investigations are not hindered.

### One pathogen, two illnesses: ST 26 has oscillated between emetic and diarrheal foodborne pathogens throughout its evolutionary history.

B. cereus was first established as the causative agent of foodborne illness in the 1950s (20, 41). Notably, prior to the 1970s, B. cereus illnesses were of the diarrheal type (i.e., toxicoinfection characterized by symptoms of watery diarrhea that onset 8 to 16 h after ingestion) (20). However, in the 1970s, a novel type of B. cereus illness, emetic intoxication, began to be reported (20). Characterized by symptoms of vomiting and nausea and a relatively short incubation time (i.e., 0.5 to 6 h), B. cereus emetic illness was first described in the United Kingdom in 1971 and was linked to the consumption of rice served at restaurants and take-away outlets (20). It has been hypothesized that emetic toxin production may confer a selective advantage (16), and the results reported here support the hypothesis that cereulide synthetase was acquired by some group III lineages relatively recently in their evolutionary histories (16). Here, we show that ST 26, which has frequently been associated with emetic foodborne illness (7, 32, 42, 43), likely first acquired cereulide synthetase and thus the ability to cause emetic illness prior to the twentieth century. This indicates that cereulide-producing B. cereus *sensu lato* may have been responsible for cryptic cases of emetic intoxication prior to the 1970s that went either undetected or unattributed to B. cereus *sensu lato*.

The temporal characterization of cereulide synthetase acquisition and loss provided here showcases that ST 26 has transitioned between an emetic and nonemetic pathogen over the course of its evolutionary history. It is essential to note that plasmid loss can occur during storage of B. cereus *sensu lato* isolates (44), which could potentially contribute to cereulide production phenotype and *ces* genotype incongruencies. While no such incongruencies were observed here, the majority of strains did not have enough phenotypic metadata available to definitively prove the absence of genotype/phenotype incongruencies. However, *ces*-negative members of ST 26 still present a relevant public health and food safety risk, since they may still be capable of causing diarrheal illness. ST 26 strain NVH 0075-95, for example, belongs to a *ces*-negative lineage that is predicted to have lost the ability to produce cereulide in the mid-20th century (i.e., between ca. 1948 and 1958). While previously shown to be incapable of producing cereulide, NVH 0075-95 produces nonhemolytic enterotoxin (Nhe), is highly cytotoxic, and was isolated from vegetable stew associated with a diarrheal outbreak in Norway (16, 45, 46). In addition, cereulide-producing strains can be high producers of diarrheal enterotoxins (8). It has been hypothesized that the simultaneous ingestion of food contaminated with cereulide alongside the cereulide- and enterotoxin-producing strains themselves may be responsible for a mixture of diarrheal and emetic symptoms among some B. cereus *sensu lato* foodborne illness cases (8), and this may partially explain why these illnesses may not always present within a strictly “emetic-versus-diarrheal” dichotomy (7, 8).

### Heterogeneous emetic phenotype presentation among diverse group III *B. cereus sensu lato* isolates can yield taxonomic inconsistencies: the emetic *B. cereus* problem.

Recent inconsistencies have arisen in the B. cereus *sensu lato* taxonomic space: B. paranthracis, a novel species proposed in 2017 (26), was found to encompass all cereulide-producing group III B. cereus *sensu lato* strains at conventional species thresholds (21). Using multiple metrics for species delineation (i.e., ANI-based genomospecies assignment, methods querying recent gene flow), we confirm that all cereulide-producing group III isolates, along with B. paranthracis and the other *ces*-negative isolates queried here (excluding outgroup genomes), belong to a single genomospecies. However, using “B. paranthracis” to describe cereulide-producing group III members is problematic, since B. paranthracis was only recently proposed as a novel species, is not well recognized outside the small B. cereus *sensu lato* taxonomic space, and hence would not typically be equated with a foodborne pathogen (21).

Referring to cereulide-producing group III lineages as emetic B. cereus, however, is also problematic. Because cereulide synthetase is often plasmid-encoded (1, 9, 10, 47), it may be possible for emetic toxin production capabilities to be lost, gained, present across multiple lineages, and absent within individual lineages (21). Here, we show that this is not just a hypothetical scenario: we observed seven cereulide synthetase gain events across group III and three loss events within ST 26 alone, indicating that cereulide synthetase loss and gain is a dynamic and ongoing process. In addition, a taxonomic label of “B. cereus” as it is applied to group III B. cereus *sensu lato* is misleading, as group III strains are not actually members of the B. cereus *sensu stricto* species, regardless of which previously proposed genomospecies threshold for B. cereus *sensu lato* is used to define species (i.e., 92.5 to 96 ANI) (7, 21, 23–26).

Taxonomic labels applied to *ces*-negative isolates interspersed among cereulide-producing group III isolates (i.e., the *ces*-negative isolates queried here) are even more ambiguous. Some *ces*-negative isolates are capable of causing diarrheal illness (16, 45, 46) and are thus relevant threats to global public health; however, there is no standardized nomenclature with which these isolates can be described. For example, the following names have been used to refer to *ces*-negative, group III strains: emetic-like B. cereus, B. cereus, group III B. cereus, B. paranthracis, or B. cereus sensu stricto/B. cereus *s.s*, although it should be noted that B. cereus *sensu stricto* is a misnomer; as mentioned previously, group III strains do not fall within the genomospecies boundary of the B. cereus *sensu stricto* type strain and thus are not actually members of the B. cereus *sensu stricto* species (12, 16, 26, 48–51).

Recently, we proposed a nomenclatural framework that can account for emetic heterogeneity among B. cereus *sensu lato* genomes through the incorporation of a standardized collection of biovar terms (21), including biovar “Emeticus.” Using this framework, all cereulide-producing members of B. cereus *sensu lato* (including emetic B. weihenstephanensis) can be referenced using the name *B.* Emeticus. All cereulide-producing group III lineages are *B. mosaicus* subspecies *cereus* biovar Emeticus (full name) or B. cereus biovar Emeticus (shortened notation), while the *ces*-negative isolates interspersed among them are *B. mosaicus* subsp. *cereus* (full name) or B. cereus (shortened notation) (21). Note that “*sensu stricto*” is not appended to these names; as mentioned above, group III B. cereus *sensu lato* lineages do not belong to the same species as group IV B. cereus *sensu stricto* type strain ATCC 14579 (7, 21).

This study is the first to offer insight into the temporal dynamics of cereulide synthetase loss and gain among group III B. cereus *sensu lato*, and it showcases the importance of accounting for emetic heterogeneity among group III lineages. As genomic sequencing grows in popularity and more group III genomes are sequenced, the estimates provided here can be further refined and improved. Furthermore, it is likely that additional cereulide synthetase loss and gain events will be observed and that previously uncharacterized emetic group III lineages will be discovered.

## MATERIALS AND METHODS

### Acquisition of group III *B. cereus sensu lato* genomes and metadata.

All genomes submitted to NCBI RefSeq (52) as a published B. cereus *sensu lato* species (21, 23–26, 53) were downloaded (*n* = 2,231; accessed 19 November 2018). FastANI v1.0 (35) and BTyper v2.3.3 (13) were used to calculate ANI values between each genome and the type strain/species reference genomes of each of the 18 published B. cereus *sensu lato* species as they existed in 2019 (see Text S1 in the supplemental material) (7). Genomes that (i) most closely resembled *B. paranthracis* and (ii) shared an ANI value of ≥95 with *B. paranthracis* were used in subsequent steps (*n* = 120), since this set of genomes contained all group III genomes that possessed cereulide synthetase-encoding genes (described in detail below). These genomes were supplemented with 30 genomes of strains isolated in conjunction with a 2016 emetic outbreak in New York State (NYS) (7), resulting in 150 group III B. cereus *sensu lato* genomes (Table S1).

10.1128/mBio.01263-20.1 TEXT S1 Detailed descriptions of all methods, plus references. Download Text S1, PDF file, 0.2 MB. Copyright © 2020 Carroll and Wiedmann. 2020 Carroll and Wiedmann This content is distributed under the terms of the Creative Commons Attribution 4.0 International license.

Metadata for all 150 genomes were obtained using publicly available records, and BTyper was used to assign each genome to a ST using the PubMLST seven-gene MLST scheme (Text S1) (54). To assess the emetic potential of each genome, BTyper was used to detect cereulide synthetase genes *cesABCD* in each genome, first using default coverage and identity thresholds (70 and 50%, respectively) and then a second time with 0% coverage to confirm *cesABCD* absence (Text S1). BTyper was additionally used to detect *cesABCD* in all 2,111 B. cereus *sensu lato* genomes not included in this study, as well as to assign all genomes to a *panC* group using the typing scheme described by Guinebretiere et al. (12). All 150 genomes selected for this study were assigned to *panC* group III, and all group III genomes possessing *cesABCD* were confirmed to have been included in this study. The only other genomes that possessed *cesABCD* belonged to *panC* group VI and most closely resembled B. mycoides/B. weihenstephanensis (i.e., emetic B. weihenstephanensis) (21).

### Construction of group III *B. cereus sensu lato* maximum-likelihood phylogenies and ancestral state reconstruction.

kSNP3 v3.1 (55, 56) was used to identify (i) core and (ii) majority SNPs among the 150 genomes described above, plus one of two outgroup genomes (to ensure that choice of outgroup did not affect ancestral state reconstruction; Text S1), using the optimal *k*-mer size determined by Kchooser (*k* = 21 for both). For each of the four SNP alignments (i.e., each combination of outgroup and either core or majority SNPs), IQ-TREE v1.6.10 (57–60) was used to construct a maximum-likelihood (ML) phylogeny (Text S1).

To ensure that ancestral state reconstruction would not be affected by genomes overrepresented in RefSeq (e.g., genomes confirmed or predicted to have been derived from strains isolated from the same outbreak), potential duplicate genomes were removed using isolate metadata and by assessing clustering in the phylogenies described above. One representative genome was selected from clusters that likely consisted of duplicate genomes and/or isolates derived from the same source. For example, this procedure reduced 30 closely related isolates from an outbreak (7) to one isolate. Overall, this approach yielded a reduced, dereplicated set of 71 genomes (Table S1). kSNP3 and IQ-TREE were again used to identify core and majority SNPs and construct ML phylogenies among the set of 71 dereplicated genomes, plus each of the two outgroup genomes, as described above, but with *k* adjusted to the optimal *k*-mer size produced by Kchooser (*k* = 23 for both).

To estimate ancestral character states of internal nodes in the group III phylogeny as they related to cereulide production (i.e., whether a node represented an ancestor that was *ces*-positive or *ces*-negative), the presence or absence of *ces* within each genome was treated as a binary state. Each of the four phylogenies constructed using the dereplicated set of 71 genomes described above was rooted at its respective outgroup, and stochastic character maps were simulated on each phylogeny using the make.simmap function in the phytools package in R v3.6.1 (61), the all-rates-different (ARD) model, and one of two root node priors (eight total combinations of two root node priors and four phylogenies; Text S1 and Table S2).

### Assessment of group III *B. cereus sensu lato* population structure.

Core SNPs detected among the 71 dereplicated group III genomes using kSNP3 (see “Construction of group III B. cereus *sensu lato* maximum-likelihood phylogenies and ancestral state reconstruction” above) were used as input for RhierBAPS (62) to identify clusters, using two levels. The same set of 71 genomes was used as input for PopCOGenT (downloaded 5 October 2019) to identify gene flow units and populations (Text S1) (22).

### Construction of group III *B. cereus sensu lato* ST 26 temporal phylogeny.

A recent study (63) has shown that the common practice of removing duplicate sequences to reduce a set of genomes to a set of unique sequences can lead to biases when constructing phylogenies using Bayesian methods. To minimize potential biases introduced by both the overrepresentation of genomes derived from a single outbreak (i.e., the 2016 NYS emetic outbreak) (7, 64), as well as the biases that sequence dereplication can introduce in a Bayesian context (63), the following ST 26 genomes were used (*n* = 37; Text S1): (i) all RefSeq ST 26 genomes that were not part of the 2016 NYS outbreak (*n* = 34) and (ii) three randomly selected NYS outbreak genomes (out of 30 possible). This set of 37 genomes was supplemented with nine additional ST 26 genomes submitted to RefSeq after 2018 (accessed 14 May 2020; Text S1), yielding a final set of 46 ST 26 genomes that underwent temporal phylogeny construction (i.e., the “New 2020/Select 3 NYS” isolate set; see Table S3 and Text S1 for detailed methods regarding the numerous isolate sets that were tested). Snippy v4.3.6 (65) was used to identify core SNPs among the 46 ST 26 genomes, using the closed chromosome of emetic ST 26 strain AH187 (NCBI RefSeq accession no. NC_011658.1) as a reference genome (Text S1). Gubbins v2.3.4 (66) was used to remove recombination from the resulting alignment, and snp-sites (67) was used to obtain core SNPs among the 46 genomes. IQ-TREE was used to construct a ML phylogeny, and TempEst v1.5.3 (68) and LSD2 v1.4.2.2 (69) were used to assess the temporal signal of the resulting phylogeny (Table S3 and Text S1).

Using the ST 26 core SNP alignment as input, BEAST v2.5.1 (70, 71) was used to construct a tip-dated phylogeny (Text S1). The Standard_TVMef nucleotide substitution model implemented in the SSM package (72) was used with 5 Gamma categories, and an ascertainment bias correction was applied to account for the use of solely variant sites (Text S1) (73). A (i) relaxed lognormal molecular clock (74) with an initial clock rate of 3.92 × 10^−8^ substitutions/site/year (75) and a broad lognormal prior on the ucldMean parameter and (ii) Coalescent Bayesian Skyline population model (76) were used, since this was the optimal clock/population model combination selected using stepping stone sampling (77) (Table S4 and Text S1).

Five independent runs using the model described above were performed, using chain lengths of at least 100 million generations, sampling every 10,000 generations. For each independent replicate, Tracer v1.7.1 (78) was used to ensure that each parameter had mixed adequately with 10% burn-in, and LogCombiner-2 was used to combine log and tree files from each independent run (Text S1). Tracer was used to construct a Coalescent Bayesian Skyline plot (see Fig. S19 [https://doi.org/10.6084/m9.figshare.c.5057276.v1]), and TreeAnnotator-2 (79) was used to produce a maximum clade credibility tree from the combined tree files, using median node heights (Text S1).

### Cereulide synthetase ancestral state reconstruction for ST 26 genomes.

Ancestral state reconstruction as it related to cereulide production was performed using the temporal ST 26 phylogeny as input (see section “Construction of group III B. cereus *sensu lato* ST 26 temporal phylogeny” above). Stochastic character maps were simulated on the phylogeny using the make.simmap function, the ARD model, and one of three priors on the root node (Table S5 and Text S1).

### Evaluation of the influence of reference genome selection on ST 26 phylogenomic topology.

To determine whether choice of reference genome affected ST 26 phylogenomic topology, SNPs were identified among 64 ST 26 genomes using four different reference-based SNP calling pipelines, chosen for their ability to utilize assembled genomes or both assembled genomes and Illumina reads as input: (i) BactSNP v1.1.0 (80), (ii) Lyve-SET v1.1.4g (81), (iii) Parsnp v1.2 (82), and (iv) Snippy v4.3.6. For alignments produced using BactSNP and Snippy, Gubbins v2.3.4 (66) was used to filter out recombination events; for Parsnp, PhiPack (83) was used to remove recombination (Text S1).

Each of four SNP calling pipelines was run six separate times, each time using one of six emetic group III reference genomes (Table 1 and Text S1). The tested reference genomes represented all available group III STs in which *cesABCD* were detected. For each SNP calling pipeline, the phylogeny constructed using SNPs identified with emetic ST 26 strain AH187 as a reference genome was treated as a reference tree, since this genome was closely related to all ST 26 isolates in the study and has previously been shown to serve as an adequate reference genome for ST 26 (7). For each of four SNP calling pipelines, the Kendall-Colijn (84, 85) test described by Katz et al. (81) was used to compare the topology of each tree to the pipeline’s respective AH187 reference phylogeny, using midpoint-rooted trees, a lambda value of 0 (to give weight to tree topology, rather than branch lengths), and a background distribution of 100,000 random trees (Text S1) (81). The Kendall-Colijn test procedure described above was then repeated for each pair of phylogenies, using the pipeline’s respective AH187 phylogeny as the query phylogeny. Pairs of trees were considered to be more topologically similar than would be expected by chance (81) if a significant *P* value resulted after a Bonferroni correction was applied (*P* < 0.05).

### Data availability.

Figures S1 to S19 have been deposited in FigShare (https://doi.org/10.6084/m9.figshare.c.5057276.v1). Accession numbers for all isolates included in this study are available in Table S1 in the supplemental material. BEAST 2 XML files, ancestral state reconstruction code, and phylogenies are available at https://github.com/lmc297/Group_III_bacillus_cereus.
