# Supplementary material for: Cereulide Synthetase Acquisition and Loss Events within the Evolutionary History of Group III Bacillus cereus Sensu Lato Facilitate the Transition between Emetic and Diarrheal Foodborne Pathogens
Source: mBio. 2020 Aug 25;11(4):e01263-20. doi: 10.1128/mBio.01263-20 (PMC7448271; doi:10.1128/mBio.01263-20)
Supplement: TEXT S1 [file mBio.01263-20-s0001.pdf]

Cereulide synthetase acquisition and loss events within the evolutionary history of Group III *Bacillus cereus sensu lato* facilitate the transition between emetic and diarrheal foodborne pathogen

Supplemental Text

Detailed descriptions of all methods, plus references.

Acquisition of Group III *Bacillus cereus s.l.* genomes and metadata. All genomes submitted to the National Center for Biotechnology Information (NCBI) RefSeq (1) database under the name of a published species belonging to *B. cereus s.l.* (i.e., one of *B. albus, anthracis, cereus, cytotoxicus, luti, mobilis, mycoides, nitratireducens, pacificus, paramycoides, paranthracis, proteolyticus, pseudomycoides, thuringiensis, toyonensis, tropicus, weihenstephanensis,* or *wiedmannii*) (2-7) were downloaded (n = 2,231; accessed November 19, 2018). The one-way average nucleotide identity BLAST (ANIb) function in BTyper version 2.3.3 (8) was used to calculate ANIb values between each of the 2,231 assembled *B. cereus s.l.* genomes and genomes of each of the 18 published *B. cereus s.l.* species as they existed in 2019 (for all but *B. anthracis*, the species type strain genome was used; for *B. anthracis*, the closed chromosome of *B. anthracis* str. Ames was used, as it is the reference genome for the species and the only type strain genome was scaffolded). *B. cereus s.l.* genomes which (i) most closely resembled the *B. paranthracis* type strain genome (i.e., the highest ANIb value was produced when the genome was compared to *B. paranthracis*), and (ii) shared an ANIb value ≥ 95 with the *B. paranthracis* type strain genome were used in subsequent steps (n = 120), as this set of genomes contained all Group III *B. cereus s.l.* genomes that possessed genes encoding cereulide synthetase (described in detail below). The resulting 120 Group III *B. cereus s.l.* genomes were supplemented with an additional 30 Group III *B. cereus s.l.* genomes of strains isolated in conjunction with a 2016 emetic outbreak in New York State (9), resulting in a total of 150 Group III *B. cereus s.l.* genomes (Supplemental Table S1). FastANI version 1.0 (10) was used to confirm that all 150 genomes selected for this study (i) shared ≥ 95 ANI with the *B. paranthracis* type strain genome,

and (ii) most closely resembled the *B. paranthracis* type strain genome when compared to the 18 *B. cereus s.l.* type strain/reference genomes.

Metadata for each of the 150 Group III *B. cereus s.l.* genomes was obtained using publicly available records. First, the NCBI BioSample (11) associated with each genome assembly was queried for (i) isolation source, (ii) geographic location, and (iii) year of isolation. If any of this information was not available within the BioSample record, the BioProject linked to the BioSample was queried. If this search did not return additional metadata, any publications (e.g., research papers, genome announcements) linked to the BioProject were queried. Finally, strain names of genomes without linked publications were queried in Google to obtain possible unlinked publications or hits in additional public databases. Using metadata that resulted from these searches, each genome was assigned (i) an isolation source, (ii) a geographic location, and (iii) a year of isolation. For isolation source, genomes were categorized into one of the following groups: ANI (isolated from an animal, excluding humans), ENV (isolated from an environment not meant for human consumption), FOO (isolated directly from a food product, food ingredient, or dietary supplement with the potential for human consumption), HUM (isolated from a human), and XXX (isolated from an unknown source; Supplemental Table S1). For geographic location, isolates were grouped by their country of isolation, except for a few cases in which a major autonomous region was listed (i.e., Hong Kong), a country which no longer existed was listed (i.e., Czechoslovakia), or a country of isolation designation was not applicable (i.e., isolation occurred in the Pacific Ocean or Antarctica; Supplemental Table S1). Isolates which could not be assigned a country of isolation were given a geographic isolation designation of XX. For year of isolation, genomes of strains with an "exact" year of isolation listed in a public database or publication were assigned to that particular year (Supplemental Table S1). For

genomes for which this information was unavailable, a "maximum year of isolation" which corresponded to the year associated with the earliest appearance of the strain in a publication or public resource (e.g., database or strain collection) was assigned (Supplemental Table S1).

Each of the 150 Group III *B. cereus s.l.* genomes were additionally assigned a sequence type (ST), as well as a designation of potentially emetic or not. To assess the emetic potential of each of the 150 Group III *B. cereus s.l.* genomes, BTyper version 2.3.3 was used to detect cereulide synthetase genes *cesABCD* in each assembly, first using the default coverage and identity thresholds (70 and 50%, respectively), and a second time with 0% coverage to ensure that *cesABCD* were absent from genomes in which the genes were not detected (the only genome that was affected by this was that of one of the outbreak isolates, FSL R9-6384, which had *cesD* split on two contigs). All isolates in which any of *cesABCD* were detected possessed all four genes; these isolates were given a designation of *ces*-positive with the potential to cause emetic disease. Isolates in which *cesABCD* were not detected were given a designation of *ces*-negative. BTyper was additionally used to detect *cesABCD* in each of the 2,111 *B. cereus s.l.* genomes not included in this study, as well as to assign all *B. cereus s.l.* genomes to a *panC* group using the typing scheme described by Guinebretiere, et al (12). All 150 *B. cereus s.l.* genomes used in this study were assigned to *panC* Group III, and all Group III genomes possessing *cesABCD* were confirmed to have been included in this study. The only other genomes that possessed *cesABCD* belonged to *panC* Group VI and most closely resembled the type strain genomes of *B. mycoides*/*B. weihenstephanensis* (referred to previously as "emetic *B. weihenstephanensis*") (7). BTyper version 2.3.3 was also used to assign each genome to a ST using the seven-gene multi-locus sequence typing (MLST) scheme available in PubMLST (13). One genome (NCBI RefSeq

Accession GCF_003270025) was assigned a probable ST of 205 but had mismatches in the *gmk* and *tpi* loci; as a result, a “x” character was appended after its ST to denote this (ST205x; Supplemental Table S1).

Using metadata and typing results obtained as described above, each genome was assigned a strain name adhering to the following format: (i) isolation source, (ii) geographic location, (iii) year of isolation, (iv) ANI-assigned species (i.e., *paranthracis*, the proposed species definition in use in 2018; note that a recently published taxonomic framework proposes the use of *mosaicus*) (7), (v) MLST-assigned ST, (vi) *ces*-positive or *ces*-negative designation, and (vii) RefSeq assembly accession or Food Microbe Tracker Strain identifier (14) for genomes obtained from RefSeq or the foodborne outbreak described by Carroll et al., respectively (Supplemental Table S1) (9). The rationale for assigning each genome a particular isolation source, geographic location, or isolation year can be found in Supplemental Table S1.

Construction of Group III *B. cereus s.l.* maximum likelihood phylogenies and ancestral state reconstruction. kSNP3 version 3.1 (15, 16) was used to identify SNPs among genomes in the following data sets: (i) all 150 Group III *B. cereus s.l.* genomes described above, plus the closed RefSeq species reference genome for *B. anthracis* (*B. anthracis* str. Ames, NCBI RefSeq Accession GCF_000007845.1; this genome would be treated as an outgroup for ancestral state reconstruction), and (ii) all 150 Group III *B. cereus s.l.* genomes described above, plus the draft genome of *B. cereus s.l.* strain AFS057383 (NCBI RefSeq Accession GCF_002574215.1; this genome would also be treated as an outgroup to ensure that choice of outgroup did not affect ancestral state reconstruction). For both data sets, Kchooser was used to determine the optimal *k*-mer size ($k = 21$ for both). Alignments of (i) core and (ii) majority (i.e., detected in > 50% of all

genomes in the alignment) SNPs detected among the 150 Group III *B. cereus s.l.* genomes in this study, plus one of two outgroup genomes (i.e., either *B. anthracis* str. Ames or *B. cereus s.l.* str. AFS057383) using kSNP3 were used as input for IQ-TREE version 1.6.10 (17). For each of the four SNP alignments (i.e., each combination of outgroup and either core or majority SNPs), the optimal ascertainment bias-aware (18) nucleotide substitution model selected using ModelFinder (i.e., the model with the lowest Bayesian Information Criterion [BIC] value) was used (19), and branch support was assessed using 1,000 replicates of the ultrafast bootstrap approximation (20, 21).

To estimate ancestral character states of internal nodes in the Group III *B. cereus s.l.* phylogeny as they related to cereulide production (i.e., whether a node in the tree represents an ancestor that is more likely to be *ces*-positive or *ces*-negative), the presence or absence of *ces* within each genome was treated as a binary state. Each of the four phylogenies constructed as described above was rooted at its respective outgroup (i.e., either *B. anthracis* str. Ames or *B. cereus s.l.* str. AFS057383) using the root function in the ape package (22, 23) in R version 3.6.1 (24). Stochastic character maps were simulated on each of the four phylogenies using the make.simmap function in the phytools package (25) and the all-rates-different (ARD) model in the ape package. For each of the four phylogenies, either (i) equal root node prior probabilities for *ces*-positive and *ces*-negative states (i.e., $P(ces\ present) = 0.5$ and $P(ces\ absent) = 0.5$), or (ii) estimated root node prior probabilities for *ces*-positive and *ces*-negative states obtained using the make.simmap function were used. For each root node prior/phylogeny combination (eight total combinations of two root node priors and four phylogenies), an empirical Bayes approach was used, in which a continuous-time reversible Markov model was fitted, followed by

1,000 simulations of stochastic character histories using the fitted model and tree tip states (Supplemental Table S2). The resulting phylogenies were plotted using the densityMap function in the phytools package.

To ensure that ancestral state reconstruction would not be affected by genomes of isolates over-represented in RefSeq (e.g., genomes confirmed or predicted to have been derived from strains isolated from the same outbreak), potential duplicate genomes were removed using isolate metadata and by assessing isolate clustering in the ML phylogenies. One representative genome was selected from clusters that likely consisted of duplicate genomes and/or isolates derived from the same source. For example, this procedure reduced 30 closely related isolates from a 2016 outbreak (9) to one isolate. Overall, this approach yielded a reduced, de-replicated set of 71 Group III *B. cereus s.l.* genomes (Supplemental Table S1). kSNP and IQ-TREE were again used to identify core and majority SNPs and construct ML phylogenies among the set of 71 de-replicated genomes, plus each of the two outgroup genomes, and ancestral state reconstruction was performed as described above (for both data sets, the optimal *k*-mer size determined by Kchooser was 23).

Assessment of Group III *B. cereus s.l.* population structure. kSNP3 version 3.1 was used to identify core SNPs among the de-replicated set of 71 Group III *B. cereus s.l.* genomes, using the optimal *k*-mer size selected by Kchooser (k = 23). The set of core SNPs produced by kSNP3 was used as input for RhierBAPS (26) to identify clusters among the 71 genomes, using two clustering levels. The same set of 71 genomes was used as input for PopCOGenT (downloaded October 5, 2019) to identify gene flow among sub-populations of Group III *B. cereus s.l.* genomes (27), using Mugsy version v1r2.3 (28), PhyML version 20120412 patch 20131031 (29),

MMseqs2 version 67c04ae456664d910059dc194863451475d2e15a (30), and MUSCLE version 3.8.31 (31).

Group III *B. cereus s.l.* ST 26 isolate set construction and temporal diagnostics. A recent study (32) has shown that the common practice of removing duplicate sequences to reduce a set of genomes to a set of unique sequences can lead to biases when constructing temporal phylogenies using Bayesian methods. To minimize potential biases introduced by both the over-representation of genomes derived from a single outbreak (i.e., the 2016 emetic outbreak in New York State) (9, 33), as well as the biases that sequence de-replication can introduce within a Bayesian framework (32), three separate isolate sets were constructed in which pseudo-random numbers generated using the random module in Python3 were used to select (i) three, (ii) five, (iii) and ten random genomes from the full set of 30 emetic ST 26 genomes from a 2016 New York State (NYS) outbreak. Each randomly selected subset of isolates derived from the known outbreak (n = 3, 5, and 10) were combined with all remaining ST 26 genomes, yielding three separate isolate sets comprising a total of 37, 39, and 44 ST 26 genomes, respectively (referred to hereafter as the “Original 2018/Select 3 NYS”, “Original 2018/Select 5 NYS”, and “Original 2018/Select 10 NYS” isolate sets, respectively; Supplemental Table S3). To additionally ensure that the inclusion of four ST 26 strains with no exact year of isolation did not significantly influence phylogeny construction (Supplemental Table S1), three additional isolate sets were constructed by removing these four isolates from each of the Original 2018/Select 3 NYS, Original 2018/Select 5 NYS, and Original 2018/Select 10 NYS isolate sets (referred to as the Original 2018/Select 3 NYS No Estimated, Original 2018/Select 5 NYS No Estimated, and Original 2018/Select 10 NYS No Estimated isolate sets, each with 33, 35, and 40 ST 26

genomes, respectively; Supplemental Table S3). Finally, an isolate set comprising all 64 ST 26 isolates (including all 30 NYS outbreak isolate genomes) was constructed (referred to as the Original 2018/All NYS Outbreak isolate set, and, due to potential biases stemming from the over-representation of isolates from a single outbreak, included merely for comparative purposes; Supplemental Table S3).

The seven aforementioned isolate sets contained all ST 26 genomes available in NCBI's RefSeq Assembly database in 2018 (accessed November 19, 2018). To identify potential additional ST 26 genomes submitted to NCBI between 2018 and 2020, the most recent set of *B. cereus s.l.* genomes available in NCBI's RefSeq Assembly database were downloaded (accessed May 14, 2020); all *B. cereus s.l.* genomes with RefSeq Assembly accession numbers that were not included in the original 2018 data set (*n* = 371) were characterized using BTyper and FastANI as described above (see section "Acquisition of Group III *Bacillus cereus s.l.* genomes and metadata" above). This search yielded an additional nine genomes assigned to ST 26 and included all five *ces*-positve *B. cereus s.l.* genomes added to RefSeq between 2018 and 2020 (i.e., among genomes included in the 2020 RefSeq Assembly download but not available in the 2018 download, cereulide synthetase-encoding *cesABCD* were detected in genomes assigned to ST 26 alone; Supplemental Table S1). The original 2018 ST 26 genomes (*n* = 64) were supplemented with these nine ST 26 genomes downloaded in 2020 (Supplemental Table S1), and four additional isolate sets were constructed by randomly selecting (i) three, (ii) five, and (iii) ten NYS outbreak genomes out of 30 total (as described above; referred to hereafter as the New 2020/Select 3 NYS, New 2020/Select 5 NYS, and New 2020/Select 10 NYS isolate sets, respectively); for the fourth isolate set (iv), all five genomes with no exact year of isolation were

removed from the New 2020/Select 5 NYS isolate set to ensure that the inclusion of these five genomes did not significantly affect phylogeny construction (referred to hereafter as the New 2020/Select 5 NYS No Estimated isolate set). The four New 2020 isolate sets contained (i) 46, (ii) 48, (iii) 53, and (iv) 43 total ST 26 genomes, respectively (Supplemental Table S3).

For each of the 11 isolate sets described above, Snippy version 4.3.6 (34) was used to identify core SNPs among all ST 26 genomes included in the isolate set, using the closed chromosome of emetic *B. cereus s.l.* ST 26 str. AH187 (NCBI RefSeq Assession NC_011658.1) as a reference genome and the following software as dependencies: BWA MEM version 0.7.13-r1126 (35, 36), Minimap2 version 2.15 (37), SAMtools version 1.8 (38), BEDtools version 2.27.1 (39, 40), BCFtools version 1.8 (41), FreeBayes version v1.1.0-60-gc15b070 (42), vcflib version v1.0.0-rc2 (43), vt version 0.57721 (44), SnpEff version 4.3T (45), samclip version 0.2 (46), seqtk version 1.2-r102-dirty (47), and snp-sites version 2.4.0 (48). Depending on the isolate set, up to 32 isolates had Illumina short reads of adequate quality after trimming and adapter removal using Trimmomatic version 0.39 (49) (as determined using FastQC version 0.11.8) (50); as such, reads were used as input for these isolates, and assembled genomes were used for all remaining ST 26 strains included in the isolate set (Supplemental Table S1).

Gubbins version 2.3.4 (51) was used to remove recombination from each resulting alignment, and snp-sites was used to obtain core SNPs among all genomes included in the isolate set. For each isolate set, IQ-TREE was used to construct a ML phylogeny, using the isolate set core SNP alignment as input, the optimal ascertainment bias-aware nucleotide substitution model selected using ModelFinder, and 1,000 replicates of the ultrafast bootstrap approximation. The

temporal signal of each resulting ML phylogeny was assessed using TempEst version 1.5.3 (52) (Supplemental Table S3).

LSD2 version 1.4.2.2 (53) was additionally used to obtain ML estimates of the evolutionary rate and time to most recent common ancestor (tMRCA) for each isolate set, using (i) the ML phylogeny for each isolate set as input, (ii) dates corresponding to the year of isolation associated with each isolate, (iii) constrained mode (-c option), (iv) variances calculated according to input branch lengths (-v 1), (v) roots estimated using constrained mode on all branches (-r as), (vi) a sequence length of 5,269,030 bp (-s 5269030, the length of the chromosome of emetic *B. cereus s.l.* ST 26 str. AH187), and (vii) a confidence interval (CI) sampling number of 1,000 (-f 1000; Supplemental Table S3). When providing dates (ii) for genomes that could not be assigned an exact year of isolation, a date range was provided for the respective genome, with bounds selected corresponding to (i) a year beyond the maximum year of isolation (upper bound; see "Acquisition of *Bacillus cereus s.l.* genomes and metadata" section above), and (ii) a high-confidence minimum value (lower bound) based on available metadata (e.g., a publication reporting that a strain was isolated within a particular timeframe, but no exact year was reported for the isolate), or, if none was available, a value of 1971.0 or 1900.0 for *ces*-positive and *ces*-negative genomes, respectively (1971.0 was used for *ces*-positive genomes, as emetic "*B. cereus*" illness was only first described in 1971) (54).

Model selection for Group III *B. cereus s.l.* ST 26 isolate sets. In order to select an optimal molecular clock/population model combination for Bayesian phylogeny construction, two isolate sets were selected to undergo the stepping stone sampling (55) procedure implemented in the MODEL_SELECTION package in BEAST version 2.5.1 (56, 57) (see section "Group III *B.*

cereus s.l. ST 26 isolate set construction and temporal diagnostics" above): (i) the Original 2018/Select 3 NYS isolate set (*n* = 37), and (ii) the Original 2018/All NYS Outbreak isolate set (*n* = 64; Supplemental Table S4). For both isolate sets, the isolate set core SNP alignment was used as input, and tip dates that corresponded to the year of isolation associated with each isolate were used. For genomes that could be assigned an exact year of isolation (see section "Acquisition of Group III *Bacillus cereus s.l.* genomes and metadata" above), a fixed tip date was used (i.e., the tip date was not estimated). For genomes that could not be assigned an exact year of isolation, tip dates were estimated using a uniform distribution, with bounds selected corresponding to (i) a year beyond the maximum year of isolation (upper bound; see "Acquisition of *Bacillus cereus s.l.* genomes and metadata" section above), and (ii) a high-confidence minimum value based on available metadata (e.g., a publication reporting that a strain was isolated within a particular timeframe, but no exact year was reported for the isolate), or, if none was available, a value of 1971.0 or 1900.0 for *ces*-positive and *ces*-negative genomes, respectively (for the Original 2018/All NYS Outbreak isolate set, a lower bound of 1900 was used for *ces*-positive genomes as well, as this isolate set is likely biased due to the over-representation of isolates from a single outbreak and was included in the study solely for comparative purposes). For each isolate set, an ascertainment bias correction based on the GC content of the closed chromosome of emetic *B. cereus s.l.* ST 26 str. AH187 was used to account for the use of solely variant sites (58). For (i) the Original 2018/Select 3 NYS isolate set, combinations of (a) either a strict or relaxed lognormal molecular clock (59) and (b) either a Constant Coalescent, Coalescent Bayesian Skyline (60), or Birth-Death Skyline Serial (61) population model were tested (i.e., six clock/population model combinations; Supplemental Table S4). For (ii) the Original 2018/All NYS Outbreak isolate set, combinations of (a) either a

strict or relaxed lognormal molecular clock and (b) either a Constant Coalescent or Coalescent Bayesian Skyline model were tested, as well as a relaxed clock/Birth-Death Skyline Serial model combination (i.e., five clock/population model combinations; Supplemental Table S4). For models that relied on a Birth-Death Skyline Serial population model, a change point was introduced into the model so that the "samplingProportion" parameter could be set to 0 before the first sample date (to account for the fact that little-to-no sampling effort was made prior to the 1970s). For (ii) the Original 2018/All NYS Outbreak isolate set, an additional change point was introduced at 2014.0 to account for the over-representation of ST 26 strains isolated between 2014 and 2016 (the most recent isolation date in the isolate set). For all models, the Standard_TVMef nucleotide substitution model implemented in the SSM package (62) was used, as it was the optimal nucleotide substitution model selected for each isolate set using the modelTest function in R's phangorn (63) package (based on BIC values), along with the Gamma category count set to 5. Additionally, for all models, a lognormal prior was placed on the clockRate and ucldMean parameters for strict and lognormal relaxed molecular clock models, respectively. For all isolate set/model combinations, marginal likelihood values were obtained for each of three independent stepping stone sampling runs performed in BEAST version 2.5.1, using ten steps with chain lengths of at least ten million generations, an alpha value of 0.3, 100,000 states of pre-burn-in, and 10% burn-in (Supplemental Table S4). For both isolate sets, a combination of a relaxed lognormal molecular clock and a Coalescent Bayesian Skyline population model was selected as the optimal clock/population model combination and was thus used in subsequent steps (Supplemental Table S4).

Group III *B. cereus s.l.* ST 26 temporal phylogeny construction. A tip-dated phylogeny was constructed for each of the eight following isolate sets using BEAST version 2.5.1 (see section "Group III *B. cereus s.l.* ST 26 isolate set construction and temporal diagnostics" above; Supplemental Table S3): (i) the Original 2018/Select 3 NYS isolate set (n = 37), (ii) the Original 2018/Select 3 NYS No Estimated isolate set (n = 33), (iii) the Original 2018/Select 5 NYS isolate set (n = 39), (iv) the Original 2018/Select 10 NYS isolate set (n = 44), (v) the Original 2018/All NYS Outbreak isolate set (n = 64), (vi) the New 2020/Select 3 NYS isolate set (n = 46), (vii) the New 2020/Select 5 NYS isolate set (n = 48), (viii) the New 2020/Select 5 NYS No Estimated isolate set (n = 43).

For each isolate set, the isolate set core SNP alignment was used as input (see section "Group III *B. cereus s.l.* ST 26 isolate set construction and temporal diagnostics" above), and isolation years were used as tip dates. For genomes that could be assigned an exact year of isolation (see section "Acquisition of Group III *Bacillus cereus s.l.* genomes and metadata" above), a fixed tip date was used (i.e., the tip date was not estimated). For genomes that could not be assigned an exact year of isolation, tip dates were estimated using a uniform distribution, with bounds selected corresponding to (i) a year beyond the maximum year of isolation (upper bound; see "Acquisition of *Bacillus cereus s.l.* genomes and metadata" section above), and (ii) a high-confidence minimum value based on available metadata (e.g., a publication reporting that a strain was isolated within a particular timeframe, but no exact year was reported for the isolate), or, if none was available, a value of 1971.0 or 1900.0 for *ces*-positive and *ces*-negative genomes, respectively (lower bound; for the Original 2018/All NYS Outbreak isolate set, which was

included merely for comparative purposes, a lower bound of 1900 was used for *ces*-positive genomes as well).

For each isolate set, an ascertainment bias correction based on the GC content of the closed chromosome of emetic *B. cereus s.l.* ST 26 str. AH187 was used to account for the use of solely variant sites (58). For all isolate sets, a relaxed lognormal molecular clock (59) and Coalescent Bayesian Skyline (60) population model were used, as it was the optimal clock/population model selected via stepping stone sampling (see section "Model selection for Group III *B. cereus s.l.* ST 26 isolate sets" above). For all isolate sets except the Original 2018/All NYS Outbreak isolate set (v; included merely for comparative purposes), an initial clock rate of 3.92×10^{-8} substitutions/site/year (estimated in a previous study of anthrax-causing Group III *B. cereus s.l.* isolates) was used (64), along with a broad lognormal prior on the ucldMean parameter (in real space, $M = 1.0 \times 10^{-4}$ and $S = 3.0$), which yielded a median of 1.11×10^{-6} and 2.5 and 97.5% quantiles of 3.11×10^{-9} and 3.97×10^{-4} substitutions/site/year, respectively. For the nucleotide substitution model, the optimal substitution model selected for the isolate set using the modelTest function in R's phangorn (63) package (based on BIC values), as implemented in the BEAST2 SSM (62) package, was used, along with the Gamma category count set to 5 (Supplemental Table S3; for seven and one of eight isolate sets, this was the Standard_TVMef and Standard_TVM model, respectively).

For each of the eight isolate sets, five independent runs using the model described above (Supplemental Table S3) were performed, using chain lengths of at least 100 million generations, sampling every 10,000 generations. For each independent run, Tracer version 1.7.1 (65) was used to ensure that effective sample size (ESS) values for all parameters were sufficiently high

(ESS > 200) and that each parameter had mixed adequately with 10% burn-in. LogCombiner-2 was used to combine log and tree files for each of the five independent runs with 10% burn-in, and Tracer was again used to (i) ensure that the combined log file showcased adequate mixing with 10% burn-in, and (ii) construct a Coalescent Bayesian Skyline plot (Supplemental Figure S19). For each isolate set, the prior was additionally sampled in the absence of sequence data, and the resulting parameter distributions were compared to the respective combined log file for the isolate set in Tracer. TreeAnnotator-2 (66) was used to produce maximum clade credibility (MCC) trees from the combined tree files associated with each isolate set, using median node heights. The resulting phylogenies were annotated using FigTree version 1.4.3 (67) and the phytools (25), ggtree (68, 69), and ape (22, 23) packages in R version 3.6.1 (24).

Among all eight isolate sets that underwent Bayesian phylogeny construction, mean and median estimates for the rate.mean parameter ranged from [1.12×10^{-7}, 2.36×10^{-7}] and [1.07×10^{-7}, 2.31×10^{-7}] substitutions/site/year, respectively, with all isolate sets producing 95% highest posterior density (HPD) intervals that overlapped and contained all mean and median rate.mean estimates in their respective bounds (Supplemental Table S3). Mean and median estimates for the TreeHeight parameter ranged from [187.82, 519.59] and [173.65, 375.71] years, respectively (Supplemental Table S3). All 95% HPD intervals for the TreeHeight parameter additionally overlapped for all isolate sets, although several New 2020 data sets produced mean and median TreeHeight parameter estimates that were greater than the TreeHeight 95% HPD upper bounds produced by several Original 2018 isolate sets (Supplemental Table S3). Skyline plots of the ST 26 effective population size showcased a dramatic decrease after 2010 for all isolate sets (Supplemental Figure S19); however, this is likely a sampling artifact (e.g., possibly resulting

from the over-representation of closely related strains isolated from increased outbreak and illness monitoring efforts after 2014) and not a true recent contraction in population size (33), and should be interpreted with extreme caution. The final temporal phylogeny reported in the main manuscript was that produced by the New 2020/Select 3 NYS isolate set (n = 46) using median node heights, as the New 2020 isolate set contained several novel ST 26 genomes that were not available in RefSeq in 2018 (Supplemental Table S1), and all New 2020 isolate sets produced similar median rate.mean and TreeHeight parameter estimates (Supplemental Table S3).

Cereulide synthetase ancestral state reconstruction for ST 26 genomes. Ancestral state reconstruction as it related to cereulide production capabilities was performed using the temporal phylogeny constructed for each of eight isolate sets using Snippy, BEAST 2, LogCombiner-2, and TreeAnnotator-2 (see section "Group III *B. cereus s.l.* ST 26 temporal phylogeny construction" above) as input. Stochastic character maps were simulated on each phylogeny using the make.simmap function, the ARD model, and one of the following three priors on the root node, corresponding to the *ces*-positive and *ces*-negative state of the root node: (i) equal probability of the root node belonging to a *ces*-positive or *ces*-negative state; (ii) estimated probabilities of the root node belonging to a *ces*-positive or *ces*-negative state, obtained using the make.simmap function; and (iii) probability of the root node being in a *ces*-positive or *ces*-negative state set to 0.2 and 0.8, respectively, as the probability of the ST 26 ancestor being *ces*-negative was estimated to be between 0.78 and 0.83 (depending on the choice of outgroup) when core SNPs among all Group III *B. cereus s.l.* genomes were used for ancestral state reconstruction (see section "Construction of Group III *B. cereus s.l.* maximum likelihood

phylogenies and ancestral state reconstruction" above). An empirical Bayes approach was used, in which a continuous-time reversible Markov model was fitted, followed by 10,000 simulations of stochastic character histories using the fitted model and the tree tip states. The resulting phylogenies were plotted using the densityMap function in the phytools package. The final ST 26 ancestral state reconstruction results reported in the main manuscript were those produced by the New 2020/Select 3 NYS isolate set (n = 46) using median node heights, as the New 2020 isolate set contained several novel ST 26 genomes that were not available in RefSeq in 2018 (Supplemental Table S1), and all New 2020 isolate sets produced similar ancestral state reconstruction results (Supplemental Table S5).

Evaluation of the influence of reference genome selection on ST 26 phylogenomic topology. To determine if choice of reference genome affected the topology of the ST 26 phylogeny, SNPs were identified among 64 Group III *B. cereus s.l.* genomes which belonged to ST 26 (i.e., the "Original 2018/All NYS Outbreak" data set) using four different reference-based SNP calling pipelines, chosen for their ability to utilize assembled genomes or both assembled genomes and Illumina reads as input: (i) BactSNP version 1.1.0 (70), (ii) Lyve-SET version 1.1.4g (71), (iii) Parsnp version 1.2 (72), and (iv) Snippy version 4.3.6. For the BactSNP, Lyve-SET, and Snippy pipelines, which can utilize both Illumina reads and assembled genomes as input, Illumina reads were used for those isolates for which they were available (n = 32; Trimmomatic and FastQC were used for preprocessing, as described in section "Construction of Group III *B. cereus s.l.* ST 26 temporal phylogeny" above), and assembled genomes were used for the remaining isolates (n = 32). For Parsnp, which relies on assembled genomes as input, all 64 ST 26 genome assemblies were used as input.

For the BactSNP pipeline, all default steps were run as outlined in the manual. Gubbins was used to remove recombination events within the resulting pseudogenome alignment (pseudo_genomes_wo_ref.fa), and snp-sites was used to obtain an alignment of SNPs. For the Lyve-SET pipeline, all default steps were run as outlined in the manual. The resulting SNP alignment (out.informative.fasta) was queried using snp-sites to obtain an alignment of core SNPs. For the Snippy pipeline, steps were run as outlined above (see section "Group III *B. cereus s.l.* ST 26 temporal phylogeny construction"), with Gubbins and snp-sites used to create a core SNP alignment. For the Parsnp pipeline, core SNPs were identified using assembled genomes as input, and Parsnp's implementation of PhiPack (73) was used to remove recombination events. For each SNP alignment identified with each pipeline, IQ-TREE was used to construct a ML phylogeny using the optimal ascertainment bias-aware nucleotide substitution model selected using ModelFinder and 1,000 replicates of the ultrafast bootstrap approximation. The dist.gene function in the ape package in R was used to calculate the number of pairwise SNP differences between each genome in each alignment.

Each of the four reference-based SNP calling pipelines described above was run six separate times, each time using one of the following Group III *B. cereus s.l.* genomes as a reference: (i) the complete, closed chromosome of emetic *B. cereus s.l.* ST 26 str. AH187 (obtained from a human clinical isolate associated with a 1972 emetic outbreak in the United Kingdom, and previously shown to serve as an adequate reference genome for reference-based SNP calling among ST 26 genomes; NCBI RefSeq Accession NC_011658.1) (9); (ii) the scaffolded draft genome of emetic *B. cereus s.l.* ST 26 str. IS195 (isolated from a pigmy shrew in Poland, and less closely related to other ST 26 isolates than *B. cereus s.l.* str. AH187; NCBI

RefSeq Accession GCF_000399225.1) (74-77); (iii) the contigs of emetic *B. cereus s.l.* ST 144 str. MB.17 (isolated from food in Munich, Germany; NCBI RefSeq Accession GCF_001566445.1) (78); (iv) the contigs of emetic *B. cereus s.l.* ST 2056 str. MB.18 (isolated from food in Munich, Germany; NCBI RefSeq Accession GCF_001566385.1) (78); (v) the contigs of emetic *B. cereus s.l.* ST 869 str. MB.22 (isolated from food in Munich, Germany; NCBI RefSeq Accession GCF_001566535.1) (78); (vi) the scaffolded draft genome of emetic *B. cereus s.l.* ST 164 str. AND1407 (isolated from black currants in Denmark; NCBI RefSeq Accession GCF_000290995.1) (79, 80). This set of tested reference genomes represented all Group III STs in which cereulide synthetase-encoding genes were detected.

For each of the four SNP calling pipelines, the phylogeny constructed using SNPs identified with emetic *B. cereus s.l.* ST 26 str. AH187 as a reference genome was treated as a reference tree. The Kendall-Colijn (81, 82) test described by Katz et al. (71) was used to compare the topology of each tree constructed with SNPs identified using each of the remaining five reference genomes (emetic Group III *B. cereus s.l.* strains IS195, MB.17, MB.18, MB.22, and AND1407, representing STs 26, 144, 2056, 869, and 164, respectively) to the AH187 reference phylogeny. For each query-reference tree combination, the Kendall-Colijn test was performed using midpoint-rooted trees, a lambda value of 0 (to give weight to tree topology, rather than branch lengths), a background distribution of 100,000 random trees (71), and the following R packages: treespace (83), phangorn, ggplot2 (84), stringr (85), docopt (86), ips (87). The Kendall-Colijn test procedure described above was then repeated for each pair of phylogenies, using the pipeline's respective AH187 phylogeny as the query phylogeny. Pairs of

trees were considered to be more topologically similar than would be expected by chance (71) if a significant *P*-value resulted after a Bonferroni correction was applied ($P < 0.05$).

Data availability. Supplemental Figures S1-S19 have been deposited in FigShare (DOI: https://doi.org/10.6084/m9.figshare.c.5057276.v1). Accession numbers for all isolates included in this study are available in Supplemental Table S1. BEAST 2 XML files, ancestral state reconstruction code, and phylogenies are available at: https://github.com/lmc297/Group_III_bacillus_cereus.

References

1. Pruitt KD, Tatusova T, Maglott DR. 2007. NCBI reference sequences (RefSeq): a curated non-redundant sequence database of genomes, transcripts and proteins. Nucleic Acids Res 35:D61-5.
2. Lechner S, Mayr R, Francis KP, Pruss BM, Kaplan T, Wiessner-Gunkel E, Stewart GS, Scherer S. 1998. *Bacillus weihenstephanensis* sp. nov. is a new psychrotolerant species of the *Bacillus cereus* group. Int J Syst Bacteriol 48 Pt 4:1373-82.
3. Guinebretiere MH, Auger S, Galleron N, Contzen M, De Sarrau B, De Buyser ML, Lamberet G, Fagerlund A, Granum PE, Lereclus D, De Vos P, Nguyen-The C, Sorokin A. 2013. *Bacillus cytotoxicus* sp. nov. is a novel thermotolerant species of the *Bacillus cereus* Group occasionally associated with food poisoning. Int J Syst Evol Microbiol 63:31-40.
4. Jimenez G, Urdiain M, Cifuentes A, Lopez-Lopez A, Blanch AR, Tamames J, Kampfer P, Kolsto AB, Ramon D, Martinez JF, Codoner FM, Rossello-Mora R. 2013. Description of *Bacillus toyonensis* sp. nov., a novel species of the *Bacillus cereus* group, and pairwise genome comparisons of the species of the group by means of ANI calculations. Syst Appl Microbiol 36:383-91.
5. Miller RA, Beno SM, Kent DJ, Carroll LM, Martin NH, Boor KJ, Kovac J. 2016. *Bacillus wiedmannii* sp. nov., a psychrotolerant and cytotoxic *Bacillus cereus* group species isolated from dairy foods and dairy environments. Int J Syst Evol Microbiol 66:4744-4753.
6. Liu Y, Du J, Lai Q, Zeng R, Ye D, Xu J, Shao Z. 2017. Proposal of nine novel species of the *Bacillus cereus* group. Int J Syst Evol Microbiol 67:2499-2508.
7. Carroll LM, Wiedmann M, Kovac J. 2020. Proposal of a Taxonomic Nomenclature for the *Bacillus cereus* Group Which Reconciles Genomic Definitions of Bacterial Species with Clinical and Industrial Phenotypes. mBio 11.
8. Carroll LM, Kovac J, Miller RA, Wiedmann M. 2017. Rapid, high-throughput identification of anthrax-causing and emetic *Bacillus cereus* group genome assemblies

using BTyper, a computational tool for virulence-based classification of *Bacillus cereus* group isolates using nucleotide sequencing data. Appl Environ Microbiol doi:10.1128/AEM.01096-17.

9. Carroll LM, Wiedmann M, Mukherjee M, Nicholas DC, Mingle LA, Dumas NB, Cole JA, Kovac J. 2019. Characterization of Emetic and Diarrheal *Bacillus cereus* Strains From a 2016 Foodborne Outbreak Using Whole-Genome Sequencing: Addressing the Microbiological, Epidemiological, and Bioinformatic Challenges. Front Microbiol 10:144.
10. Jain C, Rodriguez RL, Phillippy AM, Konstantinidis KT, Aluru S. 2018. High throughput ANI analysis of 90K prokaryotic genomes reveals clear species boundaries. Nat Commun 9:5114.
11. Barrett T, Clark K, Gevorgyan R, Gorelenkov V, Gribov E, Karsch-Mizrachi I, Kimelman M, Pruitt KD, Resenchuk S, Tatusova T, Yaschenko E, Ostell J. 2012. BioProject and BioSample databases at NCBI: facilitating capture and organization of metadata. Nucleic Acids Res 40:D57-63.
12. Guinebretiere MH, Velge P, Couvert O, Carlin F, Debuyser ML, Nguyen-The C. 2010. Ability of *Bacillus cereus* group strains to cause food poisoning varies according to phylogenetic affiliation (groups I to VII) rather than species affiliation. J Clin Microbiol 48:3388-91.
13. Jolley KA, Maiden MC. 2010. BIGSdb: Scalable analysis of bacterial genome variation at the population level. BMC Bioinformatics 11:595.
14. Vangay P, Fugett EB, Sun Q, Wiedmann M. 2013. Food microbe tracker: a web-based tool for storage and comparison of food-associated microbes. J Food Prot 76:283-94.
15. Gardner SN, Hall BG. 2013. When whole-genome alignments just won't work: kSNP v2 software for alignment-free SNP discovery and phylogenetics of hundreds of microbial genomes. PLoS One 8:e81760.
16. Gardner SN, Slezak T, Hall BG. 2015. kSNP3.0: SNP detection and phylogenetic analysis of genomes without genome alignment or reference genome. Bioinformatics 31:2877-8.
17. Nguyen LT, Schmidt HA, von Haeseler A, Minh BQ. 2015. IQ-TREE: a fast and effective stochastic algorithm for estimating maximum-likelihood phylogenies. Mol Biol Evol 32:268-74.
18. Lewis PO. 2001. A likelihood approach to estimating phylogeny from discrete morphological character data. Syst Biol 50:913-25.
19. Kalyaanamoorthy S, Minh BQ, Wong TKF, von Haeseler A, Jermiin LS. 2017. ModelFinder: fast model selection for accurate phylogenetic estimates. Nat Methods 14:587-589.
20. Minh BQ, Nguyen MA, von Haeseler A. 2013. Ultrafast approximation for phylogenetic bootstrap. Mol Biol Evol 30:1188-95.
21. Hoang DT, Chernomor O, von Haeseler A, Minh BQ, Vinh LS. 2018. UFBoot2: Improving the Ultrafast Bootstrap Approximation. Mol Biol Evol 35:518-522.
22. Paradis E, Claude J, Strimmer K. 2004. APE: Analyses of Phylogenetics and Evolution in R language. Bioinformatics 20:289-90.
23. Paradis E, Schliep K. 2019. ape 5.0: an environment for modern phylogenetics and evolutionary analyses in R. Bioinformatics 35:526-528.

24. R Core Team. 2019. R: A Language and Environment for Statistical Computing, v3.6.1. R Foundation for Statistical Computing, Vienna, Austria. https://www.R-project.org/.
25. Revell LJ. 2012. phytools: an R package for phylogenetic comparative biology (and other things). Methods in Ecology and Evolution 3:217-223.
26. Tonkin-Hill G, Lees JA, Bentley SD, Frost SDW, Corander J. 2018. RhierBAPS: An R implementation of the population clustering algorithm hierBAPS. Wellcome Open Res 3:93.
27. Arevalo P, VanInsberghe D, Elsherbini J, Gore J, Polz MF. 2019. A Reverse Ecology Approach Based on a Biological Definition of Microbial Populations. Cell 178:820-834 e14.
28. Angiuoli SV, Salzberg SL. 2011. Mugsy: fast multiple alignment of closely related whole genomes. Bioinformatics 27:334-42.
29. Guindon S, Dufayard JF, Lefort V, Anisimova M, Hordijk W, Gascuel O. 2010. New algorithms and methods to estimate maximum-likelihood phylogenies: assessing the performance of PhyML 3.0. Syst Biol 59:307-21.
30. Steinegger M, Soding J. 2017. MMseqs2 enables sensitive protein sequence searching for the analysis of massive data sets. Nat Biotechnol 35:1026-1028.
31. Edgar RC. 2004. MUSCLE: multiple sequence alignment with high accuracy and high throughput. Nucleic Acids Res 32:1792-7.
32. Boskova V, Stadler T. 2020. PIQMEE: Bayesian phylodynamic method for analysis of large datasets with duplicate sequences. Molecular Biology and Evolution doi:10.1093/molbev/msaa136.
33. Lapierre M, Blin C, Lambert A, Achaz G, Rocha EP. 2016. The Impact of Selection, Gene Conversion, and Biased Sampling on the Assessment of Microbial Demography. Mol Biol Evol 33:1711-25.
34. Seemann T. 2019. Snippy: Rapid haploid variant calling and core genome alignment, v4.3.6. https://github.com/tseemann/snippy.
35. Li H, Durbin R. 2009. Fast and accurate short read alignment with Burrows-Wheeler transform. Bioinformatics 25:1754-60.
36. Li H. 2013. Aligning sequence reads, clone sequences and assembly contigs with BWA-MEM. arXiv:1303.3997.
37. Li H. 2018. Minimap2: pairwise alignment for nucleotide sequences. Bioinformatics 34:3094-3100.
38. Li H, Handsaker B, Wysoker A, Fennell T, Ruan J, Homer N, Marth G, Abecasis G, Durbin R, Genome Project Data Processing S. 2009. The Sequence Alignment/Map format and SAMtools. Bioinformatics 25:2078-9.
39. Quinlan AR. 2014. BEDTools: The Swiss-Army Tool for Genome Feature Analysis. Curr Protoc Bioinformatics 47:11 12 1-34.
40. Quinlan AR, Hall IM. 2010. BEDTools: a flexible suite of utilities for comparing genomic features. Bioinformatics 26:841-2.
41. Li H. 2011. A statistical framework for SNP calling, mutation discovery, association mapping and population genetical parameter estimation from sequencing data. Bioinformatics 27:2987-93.
42. Garrison E, Marth G. 2012. Haplotype-based variant detection from short-read sequencing. arXiv:1207.3907.

43. Cleary JG, Braithwaite R, Gaastra K, Hilbush BS, Inglis S, Irvine SA, Jackson A, Littin R, Rathod M, Ware D, Zook JM, Trigg L, De La Vega FM. 2015. Comparing Variant Call Files for Performance Benchmarking of Next-Generation Sequencing Variant Calling Pipelines. bioRxiv doi:10.1101/023754:023754.
44. Tan A, Abecasis GR, Kang HM. 2015. Unified representation of genetic variants. Bioinformatics 31:2202-4.
45. Cingolani P, Platts A, Wang le L, Coon M, Nguyen T, Wang L, Land SJ, Lu X, Ruden DM. 2012. A program for annotating and predicting the effects of single nucleotide polymorphisms, SnpEff: SNPs in the genome of *Drosophila melanogaster* strain w1118; iso-2; iso-3. Fly (Austin) 6:80-92.
46. Seemann T. 2019. samclip: Filter SAM file for soft and hard clipped alignments, v0.2. https://github.com/tseemann/samclip.
47. Li H. 2019. Seqtk: a fast and lightweight tool for processing sequences in the FASTA or FASTQ format, v1.2-r102-dirty https://github.com/lh3/seqtk.
48. Page AJ, Taylor B, Delaney AJ, Soares J, Seemann T, Keane JA, Harris SR. 2016. SNP-sites: rapid efficient extraction of SNPs from multi-FASTA alignments. Microb Genom 2:e000056.
49. Bolger AM, Lohse M, Usadel B. 2014. Trimmomatic: a flexible trimmer for Illumina sequence data. Bioinformatics 30:2114-20.
50. Andrews S. 2019. FastQC: a quality control tool for high throughput sequence data, v0.11.8. https://www.bioinformatics.babraham.ac.uk/projects/fastqc/.
51. Croucher NJ, Page AJ, Connor TR, Delaney AJ, Keane JA, Bentley SD, Parkhill J, Harris SR. 2015. Rapid phylogenetic analysis of large samples of recombinant bacterial whole genome sequences using Gubbins. Nucleic Acids Res 43:e15.
52. Rambaut A, Lam TT, Max Carvalho L, Pybus OG. 2016. Exploring the temporal structure of heterochronous sequences using TempEst (formerly Path-O-Gen). Virus Evol 2:vew007.
53. To T-H, Jung M, Lycett S, Gascuel O. 2015. Fast Dating Using Least-Squares Criteria and Algorithms. Systematic Biology 65:82-97.
54. Tewari A, Abdullah S. 2015. *Bacillus cereus* food poisoning: international and Indian perspective. J Food Sci Technol 52:2500-11.
55. Xie W, Lewis PO, Fan Y, Kuo L, Chen MH. 2011. Improving marginal likelihood estimation for Bayesian phylogenetic model selection. Syst Biol 60:150-60.
56. Bouckaert R, Vaughan TG, Barido-Sottani J, Duchene S, Fourment M, Gavryushkina A, Heled J, Jones G, Kuhnert D, De Maio N, Matschiner M, Mendes FK, Muller NF, Ogilvie HA, du Plessis L, Popinga A, Rambaut A, Rasmussen D, Siveroni I, Suchard MA, Wu CH, Xie D, Zhang C, Stadler T, Drummond AJ. 2019. BEAST 2.5: An advanced software platform for Bayesian evolutionary analysis. PLoS Comput Biol 15:e1006650.
57. Bouckaert R, Heled J, Kuhnert D, Vaughan T, Wu CH, Xie D, Suchard MA, Rambaut A, Drummond AJ. 2014. BEAST 2: a software platform for Bayesian evolutionary analysis. PLoS Comput Biol 10:e1003537.
58. Bouckaert R. 2014. Correcting for constant sites in BEAST2. https://groups.google.com/forum/#!topic/beast-users/QfBHMOqImFE. Accessed July 11, 2020.

59. Drummond AJ, Ho SY, Phillips MJ, Rambaut A. 2006. Relaxed phylogenetics and dating with confidence. PLoS Biol 4:e88.
60. Drummond AJ, Rambaut A, Shapiro B, Pybus OG. 2005. Bayesian coalescent inference of past population dynamics from molecular sequences. Mol Biol Evol 22:1185-92.
61. Stadler T, Kuhnert D, Bonhoeffer S, Drummond AJ. 2013. Birth-death skyline plot reveals temporal changes of epidemic spread in HIV and hepatitis C virus (HCV). Proc Natl Acad Sci U S A 110:228-33.
62. Bouckaert R, Xie D. 2017. SSN: Standard Nucleotide Substitution Models, http://doi.org/10.5281/zenodo.995740.
63. Schliep KP. 2011. phangorn: phylogenetic analysis in R. Bioinformatics 27:592-3.
64. Zimmermann F. 2019. Epidemiology and Ecology of *Bacillus cereus* biovar *anthracis* in Taï National Park, Côte d'Ivoire doi:http://dx.doi.org/10.17169/refubium-1460.
65. Rambaut A, Drummond AJ, Xie D, Baele G, Suchard MA. 2018. Posterior Summarization in Bayesian Phylogenetics Using Tracer 1.7. Syst Biol 67:901-904.
66. Heled J, Bouckaert RR. 2013. Looking for trees in the forest: summary tree from posterior samples. BMC Evol Biol 13:221.
67. Rambaut A. 2016. FigTree: a graphical viewer of phylogenetic trees, v1.4.3. http://tree.bio.ed.ac.uk/software/figtree/.
68. Yu G, Smith DK, Zhu H, Guan Y, Lam TT-Y. 2017. ggtree: an r package for visualization and annotation of phylogenetic trees with their covariates and other associated data. Methods in Ecology and Evolution 8:28-36.
69. Yu G, Lam TT, Zhu H, Guan Y. 2018. Two Methods for Mapping and Visualizing Associated Data on Phylogeny Using Ggtree. Mol Biol Evol 35:3041-3043.
70. Yoshimura D, Kajitani R, Gotoh Y, Katahira K, Okuno M, Ogura Y, Hayashi T, Itoh T. 2019. Evaluation of SNP calling methods for closely related bacterial isolates and a novel high-accuracy pipeline: BactSNP. Microb Genom 5.
71. Katz LS, Griswold T, Williams-Newkirk AJ, Wagner D, Petkau A, Sieffert C, Van Domselaar G, Deng X, Carleton HA. 2017. A Comparative Analysis of the Lyve-SET Phylogenomics Pipeline for Genomic Epidemiology of Foodborne Pathogens. Front Microbiol 8:375.
72. Treangen TJ, Ondov BD, Koren S, Phillippy AM. 2014. The Harvest suite for rapid core-genome alignment and visualization of thousands of intraspecific microbial genomes. Genome Biol 15:524.
73. Bruen TC, Philippe H, Bryant D. 2006. A simple and robust statistical test for detecting the presence of recombination. Genetics 172:2665-81.
74. Castiaux V, N'Guessan E, Swiecicka I, Delbrassinne L, Dierick K, Mahillon J. 2014. Diversity of pulsed-field gel electrophoresis patterns of cereulide-producing isolates of *Bacillus cereus* and *Bacillus weihenstephanensis*. FEMS Microbiol Lett 353:124-31.
75. Van der Auwera GA, Feldgarden M, Kolter R, Mahillon J. 2013. Whole-Genome Sequences of 94 Environmental Isolates of *Bacillus cereus Sensu Lato*. Genome Announc 1.
76. Swiecicka I, Fiedoruk K, Bednarz G. 2002. The occurrence and properties of *Bacillus thuringiensis* isolated from free-living animals. Lett Appl Microbiol 34:194-8.
77. Swiecicka I, De Vos P. 2003. Properties of *Bacillus thuringiensis* isolated from bank voles. J Appl Microbiol 94:60-4.

78. Crovadore J, Calmin G, Tonacini J, Chablais R, Schnyder B, Messelhausser U, Lefort F. 2016. Whole-Genome Sequences of Seven Strains of *Bacillus cereus* Isolated from Foodstuff or Poisoning Incidents. Genome Announc 4.
79. Hoton FM, Fornelos N, N'Guessan E, Hu X, Swiecicka I, Dierick K, Jaaskelainen E, Salkinoja-Salonen M, Mahillon J. 2009. Family portrait of *Bacillus cereus* and *Bacillus weihenstephanensis* cereulide-producing strains. Environ Microbiol Rep 1:177-83.
80. Biodefense and Emerging Infections (BEI) Research Resources Repository. 2019. *Bacillus cereus* Strain AND1407, NR-22159. https://www.beiresources.org/Catalog/Bacteria/NR-22159.aspx. Accessed December 24, 2019.
81. Kendall M, Colijn C. 2016. Mapping Phylogenetic Trees to Reveal Distinct Patterns of Evolution. Molecular Biology and Evolution 33:2735-2743.
82. Kendall M, Colijn C. 2015. A tree metric using structure and length to capture distinct phylogenetic signals. arXiv:1507.05211.
83. Jombart T, Kendall M, Almagro-Garcia J, Colijn C. 2017. treespace: Statistical exploration of landscapes of phylogenetic trees. Mol Ecol Resour 17:1385-1392.
84. Wickham H. 2016. ggplot2: Elegant Graphics for Data Analysis. Springer-Verlag New York.
85. Wickham H. 2019. stringr: Simple, Consistent Wrappers for Common String Operations, https://CRAN.R-project.org/package=stringr.
86. de Jonge E. 2018. docopt: Command-Line Interface Specification Language, https://CRAN.R-project.org/package=docopt.
87. Heibl C. 2008. PHYLOCH: R language tree plotting tools and interfaces to diverse phylogenetic software packages, http://www.christophheibl.de/Rpackages.html.
